# Real‐world outcomes of 18,186 metastatic solid tumor outpatients: Baseline blood cell counts correlate with survival after immune checkpoint inhibitor therapy

**DOI:** 10.1002/cam4.6645

**Published:** 2023-11-14

**Authors:** Jerome H. Goldschmidt, Lin‐Na Chou, Philip K. Chan, Liwei Chen, Nicholas Robert, Joyce Kinsey, Katherine Pitts, Matt Nestor, Edwin P. Rock, Hillard M. Lazarus

**Affiliations:** ^1^ US Oncology, Inc The Woodlands Texas USA; ^2^ Ontada Irving Texas USA; ^3^ Partner Therapeutics, Inc Lexington Massachusetts USA; ^4^ Department of Medicine, Division of Hematology and Oncology Case Western Reserve University Cleveland Ohio USA

**Keywords:** biomarkers, check point control, medical oncology, melanoma, non small cell lung cancer, renal cancer

## Abstract

**Background:**

Patient survival in advanced/metastatic melanoma, non‐small cell lung cancer (NSCLC), and renal cell carcinoma (RCC) has improved with immune checkpoint inhibitors (ICI). Biomarkers' role in prognosis and treatment has been limited by conflicting trial results.

**Methods:**

This retrospective, observational study analyzed baseline demographic, clinical, laboratory, and treatment data versus outcomes of The US Oncology Network adult outpatients. Patients with advanced/metastatic melanoma, NSCLC, or RCC treated between January 1, 2015 and November 30, 2020 were given ICI monotherapy or combination therapy with ipilimumab, pembrolizumab, nivolumab, or atezolizumab. Treatment outcomes (overall survival [OS], time to treatment discontinuation, time to next treatment) were followed longitudinally until May 31, 2021, last patient record, or date of death. Baseline blood cell counts, including absolute monocyte count (AMC), absolute lymphocyte count (ALC), monocyte‐to‐lymphocyte ratio (MLR), absolute neutrophil count (ANC), and eosinophil count, were subdivided into quintiles for univariate and multivariable Cox regression analyses.

**Results:**

Data from 18,186 patients with advanced/metastatic melanoma (*n* = 3314), NSCLC (*n* = 12,416), and RCC (*n* = 2456) were analyzed. Better OS correlated with increased baseline serum albumin concentration, increased eosinophil and lymphocyte counts, and Western United States physician practice location. Decreased OS correlated with increased AMC, MLR, ANC, age, and worse Eastern Cooperative Oncology Group performance status.

**Conclusions:**

To our knowledge, this study is the largest to date to associate baseline survival indicators and outcomes in outpatients with advanced/metastatic melanoma, NSCLC, or RCC and receiving ICIs. Results may inform disease‐specific prognostic models and help providers identify patients most likely to benefit from ICI therapy.

## Graphical abstract

This retrospective observational study of real‐world baseline demographic, clinical, laboratory, and treatment data from 18,186 US oncology outpatients who have advanced or metastatic melanoma, non‐small cell lung cancer, or renal cell carcinoma showed blood cell counts correlated with overall survival in immune checkpoint inhibitor therapy.

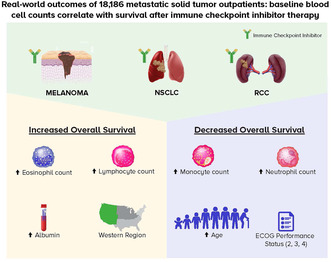

**Lay Summary**

This study analyzed data collected from an electronic health record database of 18,186 patients. Routine pre‐treatment blood test data was assessed for correlation with survival for patients with advanced skin, lung, or kidney tumors receiving treatment that stimulates the person’s immune system to fight cancer. Patient information came from The US Oncology Network community clinics between January 1, 2015 and May 31, 2021. Results showed blood cell count tests corresponded with patient survival. Improved or worsened survival depended on white blood cell type/count. More research is needed to learn how this testing can help health care providers improve patient outcomes.

## 1 INTRODUCTION

Of the estimated 1.9 million new cancer diagnoses anticipated in the United States (US) for 2022, a large proportion is expected to be melanoma (99,780), lung cancer (236,740), and renal cancer (79,000).^1^ For patients with metastatic disease, only 31.9% (melanoma), 7.0% (lung), and 15.3% (renal) are likely to survive for 5 years.^1^ Better indicators are needed to help providers understand the relationship between patient and disease in order to select optimal therapeutic regimens, ideally those with broad relevance to multiple tumor types.

Melanoma, renal cell carcinoma (RCC), and possibly non‐small cell lung cancer (NSCLC) are highly immunogenic tumors.^2^, ^3^ This attribute led to development of immune checkpoint inhibitors (ICIs) (e.g., ipilimumab, pembrolizumab, nivolumab, and atezolizumab) as standard of care for all three tumor types particularly in the setting of metastatic disease where effective therapies were limited.^4^, ^5^, ^6^ Despite improved overall survival results, many patients receiving ICIs do not respond, relapse, suffer from immune‐related adverse events, and/or incur high costs of therapy, or a combination of these factors.^7^, ^8^ Established survival indicators may help providers mitigate these treatment risks.^9^, ^10^ Serum albumin concentration, age, and Eastern Cooperative Oncology Group performance status (ECOG PS) are known to correlate with patient survival and are determined in routine care. Often a panel of predictors, such as the International Metastatic Renal Cell Carcinoma Database Consortium (IMDC) criteria for RCC, is used in determining prognosis.^11^ Investigators have also researched other potential indicators including tumor PD‐L1 expression, epidermal growth factor receptor (EGFR) mutation status, anaplastic lymphoma kinase (ALK) status, c‐ros oncogene 1 (ROS_1_) status, B‐Raf proto‐oncogene (BRAF) mutation status, mesenchymal epithelial transition (MET) status, and KRAS proto‐oncogene (KRAS) mutation status; trials have yielded conflicting results.^12^, ^13^, ^14^ Finding reliable survival indicators is a key component of precision medicine which is needed to optimize patient outcomes.

Biomarker research applicable to immunogenic tumor ICI therapy is ongoing.^2^ Beyond PD‐L1 status in NSCLC, no clinically validated prognostic biomarkers inform ICI therapeutic decision making.^13^, ^14^ Recent data suggest weak utility of PD‐L1 tumor status as malignancies with low PD‐L1 expression may still respond to ICI therapy, albeit at a lower rate, perhaps due to heterogeneity of PD‐L1 expression throughout tumor tissue or differing PD‐L1 expression detection methods and cut‐off criteria.^2^ Studies of blood cell counts and their effects on survival have yielded conflicting results, and publication bias might favor false positive signal reports from small studies.^15^, ^16^, ^17^, ^18^, ^19^, ^20^, ^21^, ^22^, ^23^, ^24^, ^25^, ^26^, ^27^, ^28^, ^29^, ^30^, ^31^ For example, increased blood absolute monocyte count (AMC) correlated with decreased overall survival for patients with melanoma receiving ICI therapy,^15^, ^20^, ^26^ but not for those diagnosed with RCC.^17^ Conversely, AMC correlated with variable survival in NSCLC across different studies.^18^, ^25^, ^30^ Despite numerous studies it remains uncertain whether individual blood cell counts^20^, ^23^, ^26^, ^28^, ^32^ or their ratios^29^, ^31^, ^33^, ^34^ reliably correlate with patient survival. In this study we retrospectively examined baseline patient demographic, clinical, laboratory, and treatment history characteristics across a large US‐based population of outpatients who had advanced/metastatic melanoma, NSCLC, or RCC that received ICI therapy. We sought to determine how routinely collected baseline blood cell counts can inform patient survival and treatment response.

## 2 METHODS

### 2.1 Patients

The study population was determined from abstracted data from medical records of US adult community oncology outpatients. Electronic health record (EHR) data were drawn from practices within The US Oncology Network or from associated clinics that use the iKnowMed™ (iKM) EHR. The US Oncology Network includes a large community of 1400 affiliated, qualified physicians operating in over 500 sites across the US. Patient demographics, clinical data, laboratory data, and treatment history were collected. All data were handled in compliance with the Health Insurance Portability and Accountability Act (HIPAA) and the Health Information Technology for Economic and Clinical Health Act (HITECH). The study protocol was granted an exemption and waiver of informed consent under 45 CFR 46.104 by The US Oncology Institutional Review Board (IRB), IRB# 20‐015E.

### 2.2 Inclusion criteria

Patients included were at least 18 years of age, had advanced or metastatic melanoma, NSCLC, or RCC between January 1, 2015, to November 30, 2020 (“the study identification period”), and received a qualifying ICI monotherapy or a combination ICI regimen of ipilimumab, pembrolizumab, nivolumab, or atezolizumab, with or without concomitant targeted therapy or chemotherapy, within those dates. Advanced or metastatic disease was defined during EHR abstraction by the following criteria: documentation of stage III or IV disease, TNM stage of M = 1, recorded metastases location, reference to metastatic disease status, or a numbered line of therapy (LOT) and/or a LOT with references to “metastatic.” Patient data must have included at least two clinic visits and included baseline blood cell counts obtained within 30 days prior to starting ICI therapy.

### 2.3 Exclusion criteria

Patients were excluded if enrolled in clinical trials of investigative medication therapy or received treatment for another documented primary cancer during the observation period. Further exclusions were considered upon medical review for patients with other concurrent cancers, or if combination treatment contained <5 patients.

### 2.4 Data collection

Data were obtained via programmed database abstraction queries of structured fields within the EHR. Patient treatment outcomes were followed longitudinally until May 31, 2021, last patient record, or date of death, whichever occurred first. Mortality data were obtained from the US Department of Commerce National Technical Information Service / Social Security Association: Limited Access Death Master File (https://dmf.ntis.gov/), if not found in the EHR. Missing (random) data were assumed to be unavailable in our fixed dataset which reflects individual/variable EHR documentation practices.

### 2.5 Outcomes

Outcomes included overall survival (OS), time to treatment discontinuation (TTD), and time to next treatment (TTNT). OS was calculated as the interval from first date of initiating a qualifying ICI regimen until date of death from any cause. Patients that did not die within the study observation period were censored on the study end date or last visit date available in the dataset, whichever occurred first. TTD was defined as the interval between the index ICI treatment initiation date and treatment discontinuation for any cause, including any treatment interruptions <90 days. Patients that did not discontinue ICI treatment during the study observation period were censored on the study end date or last visit date available in the dataset, whichever occurred first. Patients who did not receive a next treatment and were alive at end of study were censored on the study end date, or last available visit date, whichever occurred first. TTNT was defined as the interval between the index ICI treatment initiation and initiation of the next ICI treatment or death. Patients that did not receive a next treatment and were alive on the study end date were censored on the study end date, or last available visit date, whichever occurred first.

### 2.6 Statistical analysis

**FIGURE 1:**
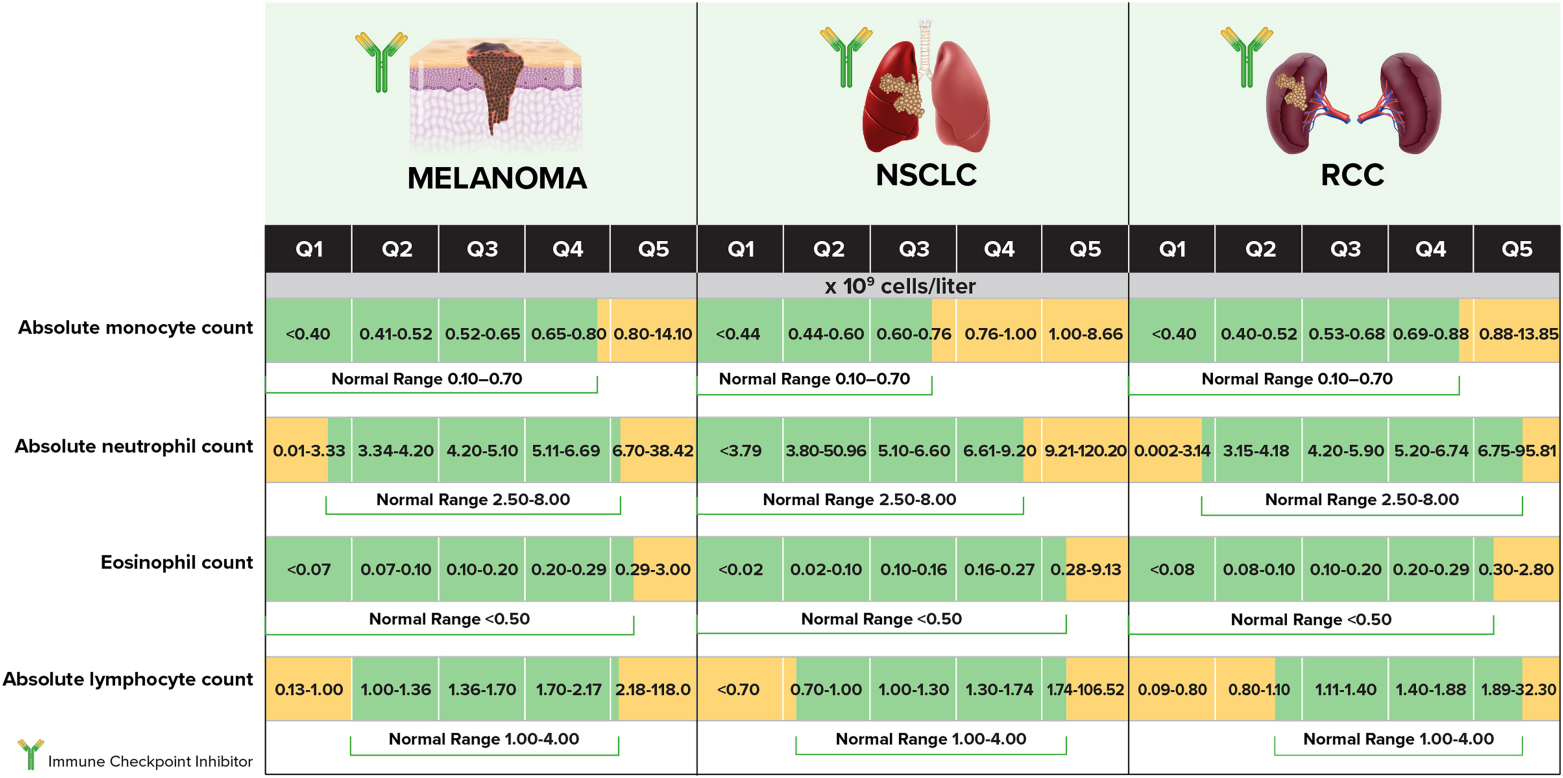
Normal range of blood counts by quintile. Normal range depicted in green. Monocyte‐to‐lymphocyte ratio (MLR) not shown as no normal range exists. NSCLC, non‐small cell lung cancer; RCC: renal cell carcinoma.

Statistical analyses were conducted using SAS® v9.4; *p*‐values <0.05 were considered statistically significant. AMC, absolute lymphocyte count (ALC), monocyte‐to‐lymphocyte ratio (MLR), absolute neutrophil count (ANC), and eosinophil count were subdivided into quintiles (Q) with Q1 including the lowest values and Q5 including the highest, based on the distribution for the sample within each tumor type (normal values in Figure 1).^35^ Quintiles were chosen to increase discriminative power among groups. All blood counts were derived by multiplication of white blood cell count and percentages of cells. Univariate and multivariable Cox regression methods were used to describe impacts of baseline biomarkers, including blood count quintiles, on OS, TTD, and TTNT by tumor type. Multivariable Cox regression models were adjusted for confounders and important survival indicators at baseline based on current practice per tumor type. Variables that had more than 50.0% of their data not documented were excluded from the multivariable Cox regression models. Multivariable Cox regression models employed a stepwise selection approach to identify covariates for multivariable models with *p* ≤ 0.25 for entry and ≤0.15 for retention. Survival indicators with significant outcome associations in univariate models and a priori variables of interest were used in multivariable modeling. Results of log‐rank tests from survival curves showed whether, for each cancer type, time‐to‐event variables (TTD, TTNT, and OS) differed by quintiles. Covariate assessment and additional statistical analysis methods are in Data S1.

## 3 RESULTS

### 3.1 Patient characteristics

**FIGURE 2:**
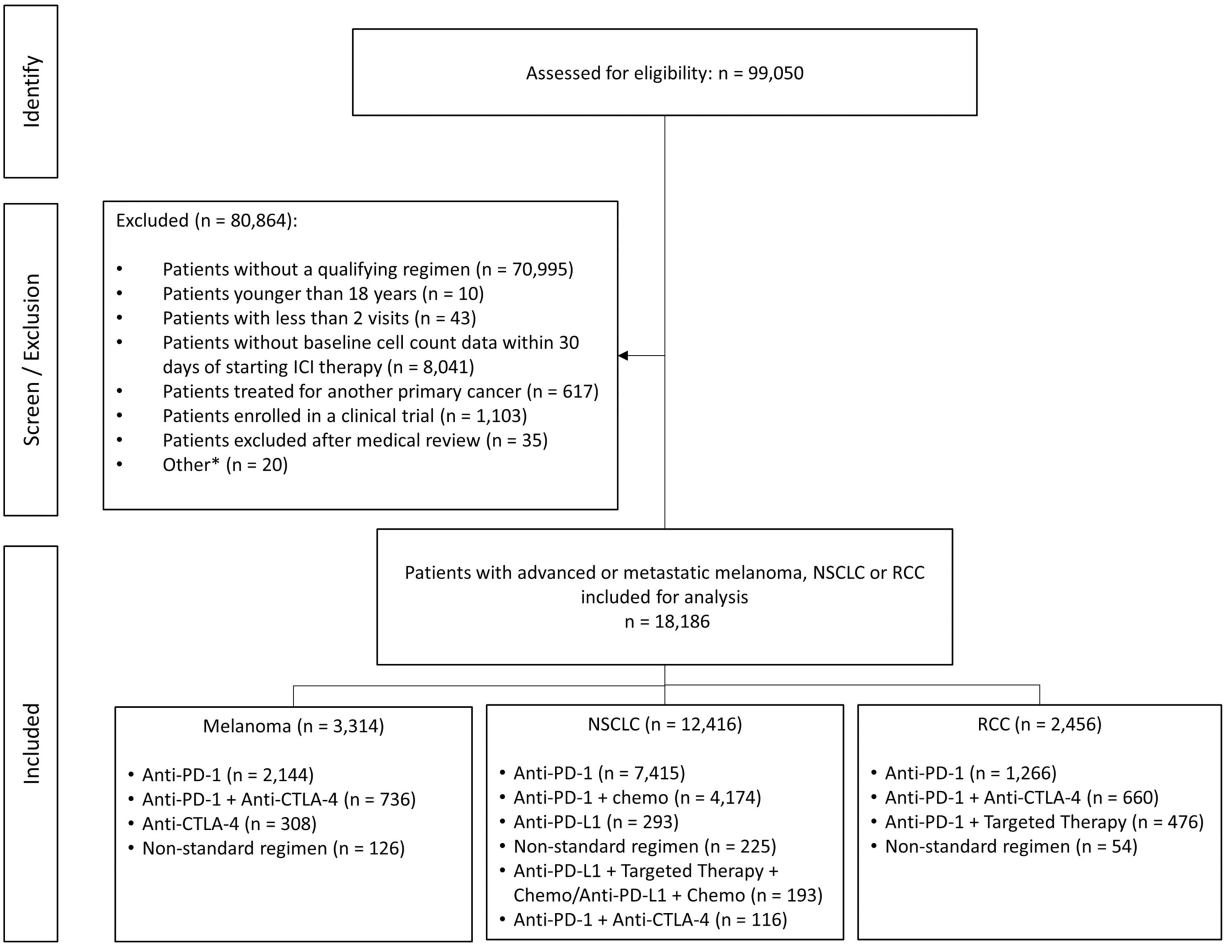
Flowchart of study design (STROBE diagram). anCTLA‐4: cytotoxic T lymphocyte‐associated antigen 4 inhibitor; anti‐PD‐1: programmed cell death protein 1 inhibitor; anti‐PD‐L1: programmed cell death ligand 1 inhibitor; ICI: immune checkpoint inhibitor; NSCLC: non‐small cell lung cancer; RCC: renal cell carcinoma. *Patients were excluded if after additional medical review by the principal investigator for patients with other concurrent cancers. Additional patients were excluded if treatment patterns were inconsistent with approved medical treatment standards or if a drug treatment class combination contained <5 patients.

We describe a large study population dataset in a US community‐based setting that illustrates relationships among baseline blood cell counts, ICI use, and outpatient outcomes in advanced or metastatic melanoma, NSCLC, and RCC. In total, 18,186 patients at over 500 different clinical sites were treated with ipilimumab, pembrolizumab, nivolumab, or atezolizumab either as monotherapy or combination therapy (Figure 2). Patients were grouped by tumor type, including melanoma (*n* = 3314), NSCLC (*n* = 12,416), and RCC (*n* = 2456). Baseline demographic, clinical, biomarker, laboratory, and drug treatment data were stratified by tumor type (Table 1; Data S1). All patients had advanced or metastatic disease at time of ICI therapy. A majority of these patients had Stage IV disease at initial diagnosis (Table 1). Median (95% CI) OS per cohort was 67.80 months (59.50–not reached [NR], melanoma), 13.60 months (13.00–14.20, NSCLC), and 28.30 months (25.60–32.00, RCC). Figure 1 shows baseline blood cell quintiles subdivisions per tumor type relative to normal, low, and high values.^35^

**TABLE 1:** Patient characteristics.

Analysis variable	Melanoma	NSCLC	RCC
	*n* = 3314	*n* = 12,416	*n* = 2456
Demographics			
Age at index^a^–years, median (IQR)	67 (57, 76)	69 (62, 76)	67 (59, 74)
Gender–*n* (%)			
Female	1201 (36.24)	5991 (48.25)	719 (29.28)
Male	2113 (63.75)	6425 (51.7)	1737 (70.72)
Race–*n* (%)			
Caucasian	2849 (85.96)	9410 (75.80)	1878 (76.47)
African American	21 (0.63)	1002 (8.10)	140 (5.70)
Native American	13 (0.39)	57 (0.50)	27 (1.10)
Asian	11 (0.33)	313 (2.50)	47 (1.90)
Other^b^	24 (0.72)	101 (0.80)	20 (0.80)
Not documented	396 (11.94)	1533 (12.34)	344 (14.01)
Tobacco use–*n* (%)			
Current	331 (9.99)	2353 (18.95)	240 (9.80)
Former	1113 (33.58)	7652 (61.63)	985 (40.11)
Never	1573 (47.46)	1366 (11.00)	977 (39.78)
Not documented	297 (8.96)	1045 (8.40)	254 (10.34)
Physician practice location–*n* (%)			
West^c^	1227 (37.02)	3794 (30.55)	856 (34.85)
South^d^	944 (28.48)	4139 (33.34)	755 (30.74)
Midwest^e^	863 (26.04)	3442 (27.72)	691 (28.14)
Northeast^f^	267 (8.05)	974 (7.84)	141 (5.74)
Not documented	13 (0.39)	67 (0.50)	13 (0.50)
Urban/rural patient status–*n* (%)			
Urban	3171 (95.68)	11,917 (95.98)	2380 (96.91)
Rural	13 (0.39)	45 (0.40)	15 (0.60)
Unknown	130 (3.92)	454 (3.70)	61 (2.50)
Clinical characteristics			
Stage at initial diagnosis–*n* (%)			
Stage 0‐II	519 (15.66)	1232 (9.92)	248 (10.10)
Stage III	152 (4.59)	2 (0.02)	267 (10.87)
Stage IIIA	244 (7.36)	1054 (8.49)	NA
Stage IIIB	281 (8.48)	802 (6.46)	NA
Stage IIIC	278 (8.39)	77 (0.62)	NA
Stage IIID	15 (0.45)	NA	NA
Stage IV	1151 (34.73)	7255 (58.43)	1501 (61.12)
Not documented	674 (20.33)	1994 (16.05)	440 (17.92)
Histology–*n* (%)			
Adenocarcinoma	NA	6939 (55.89)	NA
Adenosquamous carcinoma	NA	222 (1.78)	NA
Large cell carcinoma	NA	148 (1.19)	NA
Squamous cell carcinoma	NA	2855 (22.99)	NA
Other	NA	336 (2.71)	NA
Not documented (Nonsquamous)	NA	1916 (15.43)	NA
ECOG PS at index–*n* (%)			
0–1	1808 (54.55)	6298 (50.72)	1103 (44.91)
2+	257 (7.75)	1999 (16.10)	302 (12.30)
Not documented	1249 (37.68)	4119 (33.17)	1051 (42.79)
Comorbidities^g^–*n* (%)			
Chronic pulmonary lung disease/asthma	111 (3.30)	919 (7.40)	141 (5.70)
Diabetes mellitus	125 (3.80)	470 (3.80)	123 (5.00)
Renal disease	116 (3.50)	899 (7.20)	1497 (60.95)
Liver disease	182 (5.50)	476 (3.80)	124 (5.00)
Hypertension	37 (1.10)	237 (1.90)	71 (2.90)
Depression	56 (1.70)	302 (2.40)	50 (2.00)
Follow up time–months from ICI therapy initiation to last visit date or death, median (IQR)	12.6 (4.40, 27.1)	6.9 (2.20, 15.4)	10.9 (4.00, 22.4)
Type of metastasis–*n* (%)			
Brain	189 (5.70)	1135 (9.10)	49 (2.00)
Other	743 (22.42)	4466 (35.97)	1257 (51.18)
None	2382 (71.87)	6815 (54.88)	1150 (46.82)
LOT–*n* (%)			
LOT1	2986 (90.10)	7130 (57.43)	1080 (43.97)
Beyond LOT1	328 (9.89)	5286 (42.57)	1376 (56.03)
Laboratory			
Albumin (mg/dL)			
Median (IQR) [*n*]	4 (3.70, 4.30) [3314]	3.7 (3.40, 4.10) [12416]	3.8 (3.40, 4.10) [2456]
AMC x 10^9^ cells/L			
Median (IQR) [*n*]	0.6 (0.5, 0.8) [3314]	0.7 (0.5, 0.9) [12416]	0.6 (0.4, 0.8) [2456]
ALC x 10^9^ cells/L			
Median (IQR) [*n*]	1.5 (1.1, 2.0) [3309]	1.1 (0.8, 1.6) [12400]	1.3 (0.9, 1.7) [2450]
MLR			
Median (IQR) [*n*]	0.4 (0.3, 0.6) [3309]	0.6 (0.4, 0.9) [12398]	0.5 (0.3, 0.7) [2450]
ANC x 10^9^ cells/L			
Median (IQR) [*n*]	4.6 (3.6, 6.2) [3145]	5.8 (4.1, 8.4) [11489]	4.7 (3.4, 6.3) [2346]
NLR			
Median (IQR) [*n*]	2.9 (2.0, 4.7) [3140]	5.1 (3.1, 9.0) [11473]	3.6 (2.4, 5.6) [2340]
Eosinophil count x 10^9^ cells/L			
Median (IQR) [*n*]	0.1 (0.1, 0.2) [3126]	0.1 (0.0, 0.2) [11509]	0.1 (0.1, 0.2) [2332]
Drug treatment class–*n* (%)			
Anti‐PD‐1 monotherapy^h^	2144 (64.70)	7415 (59.72)	1266 (51.55)
Anti‐CTLA‐4 monotherapy^i^	308 (9.29)	0 (0.00)	0 (0.00)
Anti‐PD‐L1 monotherapy^j^	0 (0.00)	293 (2.36)	0 (0.00)
Anti‐PD‐1 + anti‐CTLA‐4^k^	736 (22.21)	116 (0.93)	660 (26.87)
Anti‐PD‐1 + chemotherapy^l^	0 (0.00)	4174 (33.62)	0 (0.00)
Anti‐PD‐1 + targeted^m^	0 (0.00)	0 (0.00)	476 (19.38)
Anti‐PD‐L1 + targeted^m^ + chemotherapy/anti‐PD‐L1 + chemotherapy^n^ ^,^ ^o^	0 (0.00)	193 (1.55)	0 (0.00)
Non‐standard immunotherapy regimen	126 (3.80)^p^ ^,^ ^q^ ^,^ ^r^ ^,^ ^s^	225 (1.81)^p^ ^,^ ^m^ ^,^ ^t^ ^,^ ^u^ ^,^ ^v^	54 (2.20)^w^ ^,^ ^y^ ^,^ ^x^

Abbreviations: ALC, absolute lymphocyte count; AMC, absolute monocyte count; ANC, absolute neutrophil count; CTLA‐4, cytotoxic T‐lymphocyte‐associated antigen 4; ECOG PS, Eastern Cooperative Oncology Group performance status; IQR, interquartile range; LOT1, first line of therapy; MLR, monocyte‐to‐lymphocyte ratio; NA, not applicable; NLR, neutrophil‐to‐lymphocyte ratio; NSCLC, non‐small cell lung cancer; PD‐L1, programmed death ligand 1; RCC, renal cell carcinoma.

^a^ Index: the first date of initiating a qualifying treatment after diagnosis (monotherapy or combination therapy of ipilimumab, pembrolizumab, nivolumab or atezolizumab).

^b^ The other category contains Hispanic/Latino.

^c^ Arizona, Colorado, Idaho, Montana, Nevada, New Mexico, Utah, Wyoming, California, Oregon, and Washington.

^d^ Florida, Georgia, Maryland, North Carolina, South Carolina, Virginia, Washington D.C., West Virginia, Alabama, Kentucky, Mississippi, Tennessee, Arkansas, Louisiana, Oklahoma, and Texas.

^e^ Illinois, Indiana, Michigan, Ohio, Wisconsin, Iowa, Kansas, Minnesota, Missouri, Nebraska, North Dakota, and South Dakota.

^f^ Connecticut, Maine, Massachusetts, New Hampshire, Rhode Island, Vermont, Pennsylvania, New Jersey, New York, and Delaware.

^g^ Comorbidities >1% included in table.

^h^ Nivolumab monotherapy or pembrolizumab monotherapy.

^i^ Ipilimumab monotherapy.

^j^ Atezolizumab monotherapy.

^k^ Nivolumab + ipilimumab.

^l^ Nivolumab + chemotherapy, pembrolizumab + chemotherapy.

^m^ Targeted: bevacizumab, cyramza, sotorasib, erlotinib, afatinib, gefitinib, osimertinib, dacomitinib, amivantamab, mobocertinib, necitumumab, crizotinib, ceritinib, alectinib, brigatinib, lorlatinib, lorlatinib, entrectinib, dabrafenib, trametinib, selpercatinib, pralsetinib, capmatinib, tepotinib, larotrectinib, entrectinib.

^n^ Atezolizumab + targeted + chemotherapy.

^o^ Atezolizumab + chemotherapy.

^p^ Nivolumab + targeted or pembrolizumab + targeted.

^q^ Ipilimumab + nivolumab + targeted.

^r^ Targeted: vemurafenib, dabrafenib, encorafenib, trametinib, cobimetinib, binimetinib, imatinib, and nilotinib.

^s^ Ipilimumab + targeted.

^t^ Nivolumab + targeted + chemotherapy, pembrolizumab + targeted + chemotherapy.

^u^ Ipilizumab + nivolumab + chemotherapy.

^v^ Atezolizumab + targeted.

^w^ Targeted: sunitinib, sorafenib, pazopanib, cabozantinib, lenvatinib, bevacizumab, axitinib, tivozanib, belzutifan, temsirolimus, and everolimus.

^y^ Ipilizumab + nivolumab + targeted.

^x^ Atezolizumab monotherapy.

### 3.2 In univariate analysis, longest median OS was associated with lower baseline AMC, ANC, and MLR, and higher baseline eosinophil cell count

**FIGURE 3:**
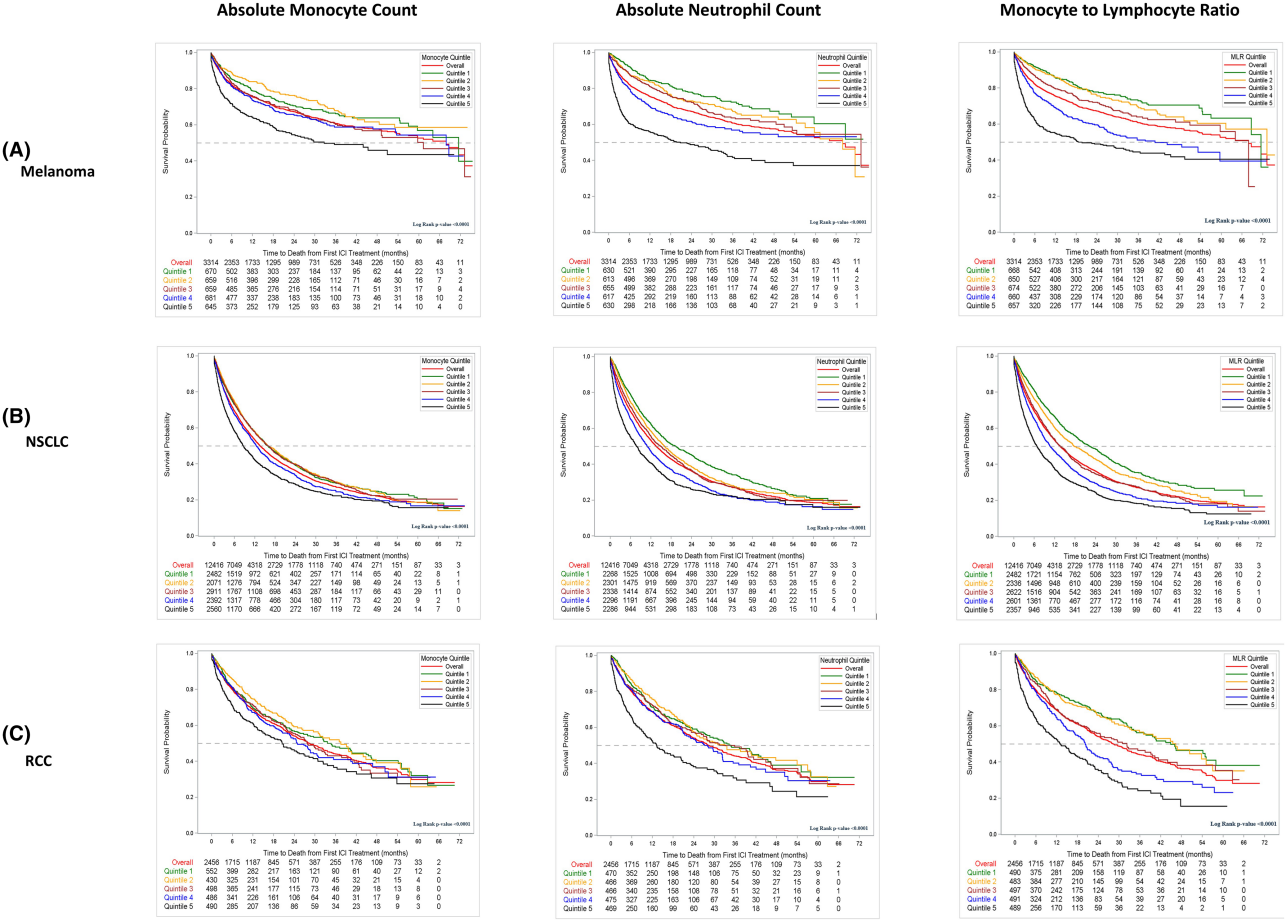
Blood cell counts correlate with overall survival. See Figure 1 for quintile values and normal lab correlates. MLR, monocyte‐to‐lymphocyte ratio; NSCLC, non‐small cell lung cancer; RCC, renal cell carcinoma.

Baseline blood cell counts were divided into quintiles (Q1–Q5), and survival curves were generated by quintile for cell counts by tumor type (Figures 1 and 3; Data S1). All quintiles (Q2‐5) were compared to Q1 to evaluate inter‐quintile relationships. For all cohorts, longest median OS was seen for lower AMC, ANC, and MLR quintiles. Conversely, median OS was longest across all cohorts for patients with higher eosinophil counts (Q5). Detailed results for AMC, ANC, MLR, and eosinophil count are in Data S1.

### 3.3 In multivariable analysis, known and investigational baseline characteristics are associated with OS in all three tumor types

**FIGURE 4:**
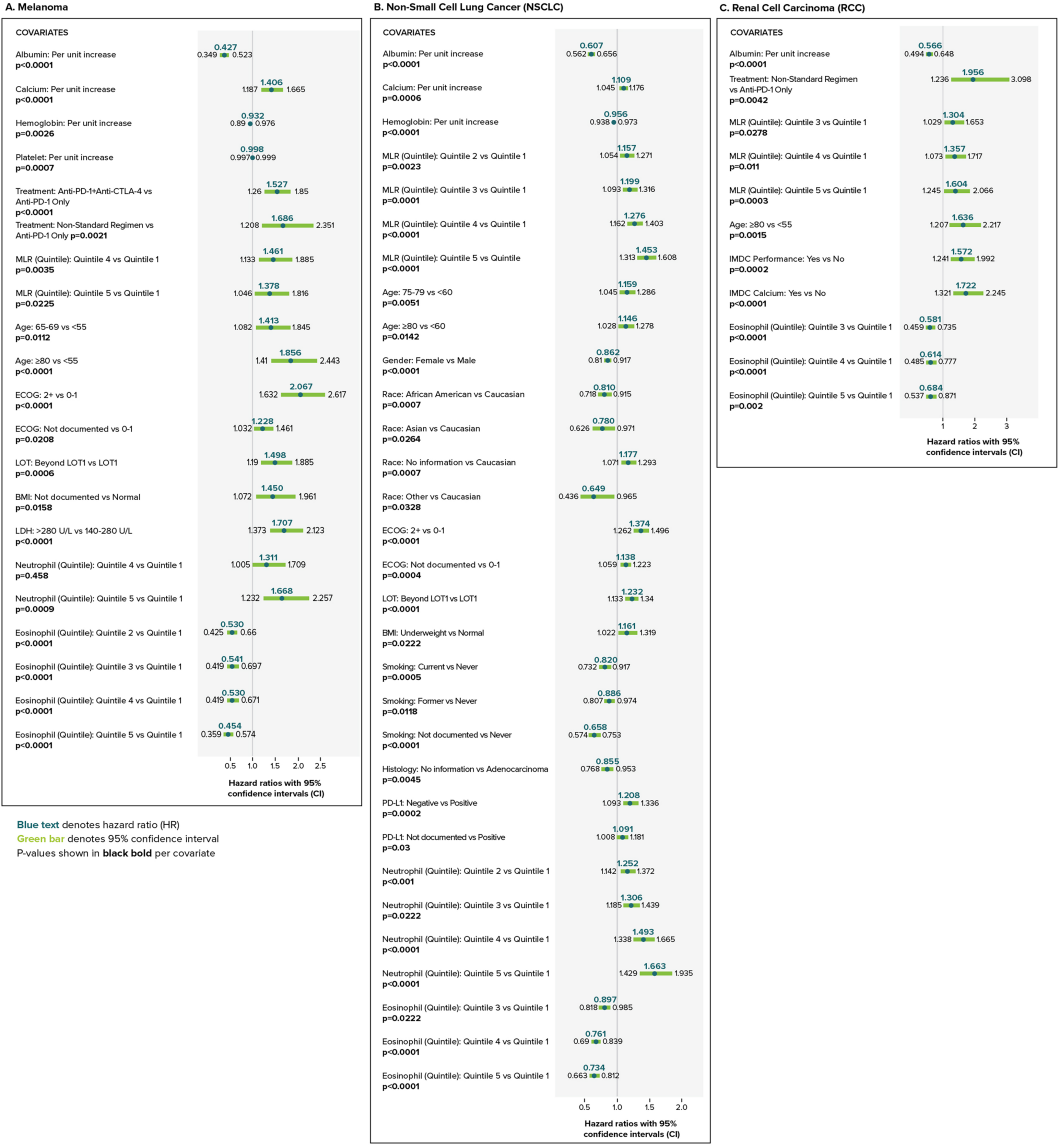
Forest plots of adjusted hazard ratios on overall survival by tumor type. Hazard ratios in blue; 95% confidence intervals in green; *p*‐values shown per covariate. Additional covariate of interest data correlated with longer or decreased OS can be found in Data S1. BMI, body mass index; CTLA‐4, cytotoxic T‐lymphocyte‐associated antigen 4; ECOG, Eastern Cooperative Oncology Group; IMDC, International Metastatic RC Database Consortium; LDH, lactate dehydrogenase; LOT, line of therapy; MLR, monocyte‐to‐lymphocyte ratio; PD‐1, programmed cell death protein‐1; PD‐L1, programmed cell death ligand‐1.

In multivariable analysis, baseline characteristics associated with OS across melanoma, NSCLC, and RCC included serum albumin concentration, age, region, eosinophil count, MLR, and ALC (Table 2). Forest plots (Figure 4) show cohort‐specific, covariate associations with increased OS, as well as risk factors for decreased OS. Known covariates, such as high serum albumin concentration as well as investigational covariates, such as high eosinophil levels were associated with longer OS in all tumor types. Increased eosinophil count (Q5) appeared to have a greater influence on OS duration for the melanoma cohort (*p* < 0.0001; HR 0.45, 95% CI 0.36–0.57) than for the NSCLC (*p* < 0.0001; HR 0.73, 95% CI 0.66–0.81) or RCC (*p* = 0.002; HR 0.68, 95% CI 0.54–0.87).

**TABLE 2:** Significant survival indicators in multivariable regression by tumor type (reflection of data from Figure 4. Forest plots of adjusted hazard ratios on overall survival by tumor type).

Confounders^a^	Melanoma	NSCLC	RCC
	*p*‐value	*p*‐value	*p*‐value
Albumin	**<0.0001**	**<0.0001**	**<0.0001**
Age	**0.0009**	**0.0034**	**<0.0001**
Region	**<0.0001**	**<0.0001**	**<0.0001**
Eosinophil (Quintile)	**<0.0001**	**<0.0001**	**<0.0001**
MLR (Quintile)	**0.0167**	**<0.0001**	**0.0032**
Lymphocyte (Quintile)	**0.0233**	**<0.0001**	**<0.0001**
Monocyte (Quintile)	NS	<0.0001	NS
Neutrophil (Quintile)	0.0188	<0.0001	NS
ECOG	<0.0001	<0.0001	NS
Line of treatment (LOT)	0.0006	<0.0001	NS
WBC (10^3^/μL)	NS	<0.0001	0.0251
Hemoglobin	0.0026	<0.0001	NS
Platelet	0.0007	<0.0001	NS
LDH	<0.0001	NS	NS
PD‐L1	NS	0.001	NS
Calcium	<0.0001	0.0006	NS
Gender	NS	<0.0001	NS
Race	NS	<0.0001	NS
BMI	NS	0.0195	NS
Smoking	NS	<0.0001	NS
IMDC performance	NS	NS	0.0009
IMDC hemoglobin	NS	NS	NS
IMDC calcium	NS	NS	0.0003

Abbreviations: BMI, body mass index; ECOG, Eastern Cooperative Oncology Group; IMDC, International Metastatic Renal Cell Carcinoma Database Consortium; LDH, lactate dehydrogenase; μL, microliter; MLR, monocyte‐to‐lymphocyte ratio; NS, not significant; NSCLC, non‐small cell lung cancer; PD‐L1, programmed death ligand‐1; RCC, renal cell carcinoma; WBC, white blood cell.

^a^ Univariate Cox regression models were first created to determine the statistical association between baseline biomarkers and outcomes (without controlling for other confounders). Confounders that were determined a priori, as well as significant confounders from the univariate Cox regression methods, were included in the multivariable Cox regression model.

Bold values denote significant survival indicators across all three tumor types.

Increased age, non‐Western physician practice location, and increased MLR were risk factors for decreased OS for all cohorts (Figure 4). Per cohort, specific physician practice location regions were more at risk as compared to the Western region of the US. Higher MLR quintiles were associated with shorter OS duration for all cohorts. Increased MLR levels (Q5) appeared to have a greater influence on OS duration for the RCC cohort (*p* = 0.0003; HR 1.60, 95% CI 1.24–2.07) than for the melanoma (*p* = 0.0225; HR 1.38, 95% CI 1.045–1.82) or NSCLC (*p* < 0.0001; HR 1.45, 95% CI 1.31–1.61) cohorts.

### 3.4 Impact of treatment agents on OS

Most patients across all cohorts received anti‐PD‐1 monotherapy (64.70% melanoma; 59.70% NSCLC; 51.50% RCC). In the melanoma cohort, anti‐CTLA‐4 monotherapy correlated with the longest median OS (95% CI) of 73.10 (68.70–NR) months (Data S1). Longest median OS in the NSCLC and RCC cohorts correlated with anti‐PD‐1 + anti‐CTLA‐4 therapy (19.20 [10.30–NR] and 30.80 [26.50–NR] months). The shortest median OS in all cohorts correlated with non‐standard immunotherapy regimens (melanoma, 7.70 [4.70–13.40] months; NSCLC, 11.80 [9.10–16.50] months; and RCC 18.10 [5.90–23.40] months).

### 3.5 Impact of treatment agents on TTD and TTNT

TTD and TTNT were evaluated as proxies for progression free survival despite limitations in their ability to attribute treatment starts and stops to toxicity versus disease progression.^36^ Further discussion of TTD, TTNT data per cell type are available in Data S1 (Figures S2–S4).

## 4 DISCUSSION

This dataset is the largest known to date to examine associations among potential baseline survival indicators in patients with advanced/metastatic melanoma, NSCLC, or RCC who were given ICIs in community oncology outpatient settings. Consistent with expectations, serum albumin concentration, ECOG PS, and age each associated with OS outcomes in univariate analyses, as reported in other studies.^9^, ^10^ These known prognostic indicators serve as positive controls to validate the relevance of this large dataset for investigation of additional survival indicators for which prior results have been less clear. While numerous prior studies of blood cell counts and their effects on survival have been executed, studies were small and have not provided clarity for this area.^15^, ^16^, ^17^, ^18^, ^19^, ^20^, ^21^, ^22^, ^23^, ^24^, ^25^, ^26^, ^27^, ^28^, ^29^, ^30^, ^31^

In our large patient series, increased baseline blood monocyte and neutrophil counts were associated with less favorable OS, whereas increased eosinophils and lymphocytes were associated with more favorable OS. These results mirror those of multiple prior studies in much smaller populations.^15^, ^18^, ^19^, ^20^, ^21^, ^23^, ^26^, ^27^, ^28^, ^29^, ^30^ Elevated blood monocyte and neutrophil counts are consistent with a persistent inflammatory state, whereas elevated baseline lymphocytes presumably facilitate the effectiveness of ICI therapy. Measuring these blood cell counts at baseline may help providers determine the clinical immune competence of their patients, i.e., baseline laboratory results may indicate the capacity of a patient to mount an ICI therapy‐induced immune response. In addition, our study and earlier studies demonstrate that elevated neutrophil‐to‐lymphocyte ratio (NLR) correlated with worse outcomes for patients on ICIs (Data S1).^29^, ^31^

Increased baseline monocytes were associated with decreased OS in all cohorts. These results might reflect increased HLA‐DR^lo/neg^ myeloid‐derived suppressor cells (MDSCs), i.e., monocytes with diminished HLA‐DR expression known to be negatively prognostic for solid tumors.^37^, ^38^, ^39^, ^40^ HLA‐DR is a major histocompatibility (MHC) class II protein found on antigen presenting cells linking innate and adaptive immune response by presenting foreign peptide antigens to antigen‐specific T lymphocytes.^37^ Reduced HLA‐DR expression on MDSCs is associated with dysregulated myelopoiesis and impairs antigen presenting cells processing of tumor‐derived peptides, yielding an impaired immune response to cancer.^38^ Elevated numbers of MDSCs are also associated with diminished ICI responses in melanoma and RCC.^37^, ^40^ Interestingly, exogenous granulocyte‐macrophage colony‐stimulating factor (GM‐CSF) upregulates HLA‐DR expression on blood monocytes, differentiates monocytes into macrophages, and maintains macrophage metabolic capacity potentially correcting primary ICI resistance.^41^, ^42^, ^43^, ^44^, ^45^, ^46^, ^47^ We believe this strategy is a very important initiative for future investigation.

In multivariable analysis, six variables (serum albumin concentration, age, region, eosinophil quintile, MLR quintile, and ALC quintile) were common survival indicators across all tumor types (Table 2). Other notable variables include calcium (melanoma); calcium, gender, race, body mass index, and smoking (NSCLC); and IMDC performance and IMDC calcium (RCC). These factors are known tumor‐specific prognostic indicators.^9^, ^10^, ^11^, ^48^

Anti‐CTLA‐4 monotherapy was associated with longer OS for patients with melanoma, whereas anti‐PD‐L1 + anti‐CLTA‐4 combination therapy were associated with longer OS for NSCLC and RCC. These findings are surprising for the melanoma cohort since anti‐PD‐1 monotherapy has been shown in previously untreated patients to improve outcomes over anti‐CLTA4 monotherapy^49^ and was more often prescribed in this dataset than anti‐CLTA‐4 monotherapy. Reasons for this disparity between recommended therapy and these real‐world data may include inherent patient selection bias (i.e., comorbidities impacting treatment selection), patient or provider preference (e.g., combination modality therapies). A non‐standard immunotherapy regimen resulted in shortest OS across cohorts and might be attributable to prior therapy failure, immune‐related adverse events, or patient preferences.

Interestingly, physician practice location (geographic region) consistently was associated with OS in this study. Patients in the Western US fared better than patients elsewhere. One explanation might be variable US regional microbiomes that influence ICI response in solid tumors based on dietary effects on microbial richness and diversity.^50^, ^51^ Alternatively, provider practice patterns, access to care, insurance coverage, health disparities, timing of diagnosis, patient lifestyle, or other environmental factors might influence regional effects on patient survival.

Strengths of this study include its large representative sample size of patients that reflects US community oncology practice trends, as opposed to interventional clinical trials with restrictive enrollment. Real‐world data studies are now being used with increased frequency as these approaches and reports appear to complement prospective trials.^52^, ^53^ Also, this study compared survival indicators across three tumor types, allowing assessment of relationships within and among tumor types. This large dataset validates associations seen in smaller, heterogenous studies of survival indicators for patient outcomes.^15^, ^18^, ^19^, ^20^, ^21^, ^23^, ^26^, ^27^, ^28^, ^29^, ^30^

Limitations follow from this study's retrospective, observational, EHR‐derived real‐world‐data design. Conclusions may be hampered by missing or incomplete staging information and other data given that EHR data are usually recorded for clinical care, not necessarily research (e.g., PD‐1 status or tumor mutational burden). We recognize therapeutic practices changed over the course of the data collection period. Concomitant medications impacting blood cell counts (i.e., corticosteroids) were not queried for analysis. Generalizability may also be limited by location distribution of The US Oncology Network practices or use of the iKM EHR, not well represented in the Northeast. Some variables of interest, such as PD‐L1 status, EGFR, ALK, ROS_1_, MET, and BRAF, were not consistently available. Structured EHR data limit progression‐free survival (PFS) calculation due to absent disease progression information. PFS measurement requires regular and reliable assessments of disease progression, infeasible in this design. Instead, proxies TTD and TTNT were used despite limitations in their ability to attribute treatment starts and stops to toxicity versus disease progression.^36^ TTD and TTNT as endpoints are further confounded by differences in the LOT at study entry, e.g., nearly all (90.1%) of the melanoma cohort was receiving ICI as first line therapy (LOT1), whereas only about half of the NSCLC cohort (57.4%) and RCC cohort (44.0%) were starting ICI therapy as LOT1 (Table 1).

This study examined baseline but not subsequent laboratory values, which may change over the course of ICI therapy. Furthermore, this study focused on initial and not subsequent ICI use, as was recently studied.^29^ Machine learning longitudinal models based on data from patients with NSCLC receiving ICI‐containing regimens showed that blood cell dynamics reflect pathologically‐assessed ICI therapy response. Interestingly, similar to our study, elevated blood eosinophils during ICI therapy correlated with therapeutic response in both metastatic and early‐stage NSCLC cohorts.

## 5 CONCLUSION

These study results may inform practitioners by building on predictive ICI response models and disease‐specific prognostic models to identify patients most likely to benefit from ICI therapy.^11^, ^28^, ^54^ Baseline blood cell counts may indicate pre‐ICI clinical immune competence and present a practical way to inform likelihood of ICI response. This research focuses on aspects of precision medicine and can provide direction for future investigations of the impact of cell count relationships, characteristics, and functional qualities on patient outcomes which might yield further opportunities to improve therapy.

## AUTHOR CONTRIBUTIONS

**Jerome H. Goldschmidt:** Methodology (equal); supervision (lead); writing – review and editing (equal). **Lin‐Na Chou:** Data curation (lead); formal analysis (lead); methodology (equal); writing – review and editing (equal). **Philip K. Chan:** Data curation (equal); formal analysis (equal); methodology (equal); project administration (lead); supervision (equal); visualization (equal); writing – review and editing (equal). **Liwei Chen:** Data curation (equal); formal analysis (equal); methodology (equal); writing – review and editing (equal). **Nicholas J. Robert:** Methodology (equal); supervision (equal); writing – review and editing (equal). **Joyce Kinsey:** Conceptualization (equal); methodology (equal); project administration (equal); writing – original draft (equal); writing – review and editing (equal). **Katherine Pitts:** Methodology (equal); writing – original draft (equal); writing – review and editing (equal). **Matt Nestor:** Conceptualization (equal); methodology (equal); project administration (equal); writing – original draft (equal); writing – review and editing (equal). **Edwin P. Rock:** Conceptualization (equal); methodology (equal); writing – original draft (equal); writing – review and editing (equal). **Hillard M. Lazarus:** Conceptualization (equal); methodology (equal); project administration (equal); supervision (equal); writing – original draft (equal); writing – review and editing (equal).

## FUNDING INFORMATION

Work was funded by Partner Therapeutics, Inc.

## CONFLICT OF INTEREST STATEMENT

Partner Therapeutics contracted Ontada to perform this study. Jerome H. Goldschmidt is an employee of Ontada and a paid consultant to Amgen and TG Therapeutics. JG serves on the speakers' bureau of Bristol Myers Squibb and G1 Therapeutics. Philip K. Chan was an employee of Ontada at the time of this study. PC owns shares of Pfizer, AstraZeneca, Inovio Pharmaceuticals, Abbott Laboratories, McKesson, and IBM stock. Lin‐Na Chou and Liwei Chen were employees of Ontada at the time of this study. Nicholas Robert is employed and holds a leadership role in Ontada. NR owns shares of McKesson and Moderna Therapeutics stock. NR acknowledges honoraria from Roche and Bristol‐Myers Squibb, and is a paid consultant of New Century Health, Bristol‐Myers Squibb, Boehringer Ingelheim, and Advi. Joyce Kinsey, Katherine Pitts, and Matt Nestor are employees of Partner Therapeutics and have stock options. Edwin P. Rock was an employee of Partner Therapeutics at the time of this study. Hillard M. Lazarus is a paid consultant to and has stock options for Partner Therapeutics and is a member of a Data Safety Monitoring Board for Bristol Myers Squibb.

## ETHICS STATEMENT

The study protocol was granted an exemption and waiver of informed consent under 45 CFR 46.104 by the US Oncology Institutional Review Board, IRB# 20‐015E.

## Supporting information


Data S1: Supplemental Methods and Results (Tables S1‐ S3 and Figures S1‐S5)

**Video S1:** Dr. Jerome Goldschmidt, Physician Investigator at The US Oncology Network, describes the baseline survival indicators found in the retrospective observational study of over 18,000 adults with advanced melanoma, non‐small cell lung cancer, or renal cell carcinoma treated with immune checkpoint inhibitors.

**Media Information:** Video 1.

## Data Availability

Requests for data will be considered upon request.
