# Supplementary material for: Real‐world outcomes of 18,186 metastatic solid tumor outpatients: Baseline blood cell counts correlate with survival after immune checkpoint inhibitor therapy
Source: Cancer Med. 2023 Nov 14;12(22):20783–97. doi: 10.1002/cam4.6645 (PMC10709745; doi:10.1002/cam4.6645)
Supplement: Supplementary file 1 — Data S1: Supplemental Methods and Results (Tables S1‐ S3 and Figures S1‐S5) [file CAM4-12-20783-s003.docx]

**Real-world outcomes of 18,186 metastatic solid tumor outpatients: baseline blood cell counts correlate with survival after immune checkpoint inhibitor therapy**

**Supplemental Methods**

**Covariate Assessment**

Univariate Cox regression models were created to determine statistical associations between baseline biomarkers and outcomes without controlling for possible confounders. Confounders that were determined *a priori*, as well as significant confounders from univariate Cox regression methods, were then tested by multivariable Cox regression modeling. Impacts of baseline patient and disease characteristics, as well as biomarkers, were evaluated for associations with overall survival (OS), time to treatment discontinuation (TTD), and time to next treatment (TTNT) per tumor type. Laboratory data were included if measured within 30 days of the “index date,” defined as initiation of qualifying treatment during study period. Comorbidity was defined by the patient problem list reported at the closest date within 30 days prior to index date.

Supplemental Table S1. Variables and Definitions

| **Variable** | **Source(s)** | **Role(s) in study** | **Period of measurement** | **Operational definition** |
| --- | --- | --- | --- | --- |
| **Demographic and patient characteristics** | | | | |
| Date of birth | iKnowMed^TM^ (iKM) structured data | Eligibility; Data linkage; Baseline characteristic | Prior medical history | Patient’s date of birth as recorded in iKM. |
| Age at index (continuous) | iKM structured data (derived) | Eligibility; Baseline characteristic | Baseline | Patient’s age (in years) at index, which was calculated as the integer of [(index date – date of birth + 1) / 365.25]. |
| Age groups at index | iKM structured data (derived) | Baseline characteristic | Baseline | The following age categories were created based on the continuous age data: <65 years; ≥65 years |
| Sex | iKM structured data | Baseline characteristic | Prior medical history | Patients were categorized as: Male; Female |
| Race | iKM structured data | Baseline characteristic | Prior medical history | Patients were categorized as: Caucasian; African American; Asian; Native American; Other (Hispanics/Latinos); Not documented |
| Height | iKM structured data | Baseline characteristic | Baseline | Patient’s height in meters. |
| Weight | iKM structured data | Baseline characteristic | Baseline | Patient’s weight in kilograms. |
| Body mass index (BMI) | iKM structured data (derived) | Baseline characteristic | Baseline | BMI = Weight (in kilograms)/(Height [in meters])^2^  Based on the resulting BMI values, patients were then categorized as underweight (BMI < 18.5), normal (BMI 18.5 – 24.9), overweight (BMI 25 – 29.9), obese (BMI ≥ 30) or Not documented (missing height or weight data). |
| Tobacco use | iKM structured data | Baseline characteristic | Baseline | Categorized as: No history of tobacco use; Current tobacco use; Former tobacco use; Not documented |
| Other cancer diagnosis | iKM structured data | Eligibility criteria | Study observation period | Patients were confirmed as not having received active treatment for another primary cancer during the study observation period. |
| Additional visits | iKM structured data | Eligibility criteria | Follow-up | Patients had to have at least 2 records of either additional visits following the index date visit and/or a record of death prior to the end of the study follow-up period. Visits are defined as physical encounters with the practice, detected by vital sign records. No distinction was made, for purposes of inclusion, between patients that had an additional US Oncology Network visit and those that had a record of death. There was no minimum or maximum requirement on time from index date to these qualifying events. |
| Practice location | iKM structured data (derived) | Baseline characteristic | Baseline | US census region of The US Oncology Network clinic where the patient received care at the index visit (date of index event):  Midwest: Illinois, Indiana, Michigan, Ohio, Wisconsin, Iowa, Kansas, Minnesota, Missouri, Nebraska, North Dakota, and South Dakota  Northeast: Connecticut, Maine, Massachusetts, New Hampshire, Rhode Island, Vermont, Pennsylvania, New Jersey, New York, and Delaware  South: Florida, Georgia, Maryland, North Carolina, South Carolina, Virginia, Washington D.C., West Virginia, Alabama, Kentucky, Mississippi, Tennessee, Arkansas, Louisiana, Oklahoma, and Texas  West: Arizona, Colorado, Idaho, Montana, Nevada, New Mexico, Utah, Wyoming, California, Oregon, and Washington State  Missing clinic values were captured in a “not documented” category. |
| Rural/urban | iKM structured data (derived) | Baseline characteristic | Baseline | Rural/urban status of The US Oncology Network clinic where the patient received care at the index visit. Rural/Urban status was defined based upon US Department of Agriculture classifications. |
| Practice size | iKM structured data (derived) | Baseline characteristic | 2015-2020 | The number of patients seen at The US Oncology Network clinic where the patient received care for his/her index visit averaged across 2015-2020. Across all patients, practice size was evaluated based on the years 2015-2020. Categories: <50 patients/ year; 50-350 patients/year; 350-1000 patients/year; ≥1000 patients/year |
| Physician melanoma, non-small cell lung cancer (NSCLC), or renal cell carcinoma (RCC) patient volume | iKM structured data (derived) | Baseline characteristic | 2015-2020 | The number of patients with overall melanoma, NSCLC, or RCC seen by the physician who provided care for the patient’s index visit averaged across 2015-2020: ≤10 patients/ year; 11-49 patients/year; ≥50 patients/year |
| Physician specialty (extracted from National Provider Identifier database) | iKM structured data | Baseline characteristic | Baseline | Specialty of the physician who provided care for the patient’s index visit: Hematology & medical oncology; Internal medicine; Other; Not documented |
| Data accessibility | iKM structured data | Eligibility criteria | Baseline | Patients whose data were accessible for research purposes were eligible for participation in the study; otherwise, patients were excluded. |
| **Disease characteristics** | | | | |
| Date of initial diagnosis of melanoma, NSCLC, or RCC | iKM structured data | Eligibility criteria, Baseline characteristic | Prior medical history | To assess melanoma, NSCLC, or RCC diagnoses that occurred prior to index, patients’ available medical history in iKM was searched. The completeness of this history could vary based on the length of disease and the time within The US Oncology Network. Records may also have been incomplete for patients with an initial diagnosis that occurred outside of The US Oncology Network.  Diagnosis was determined through a review of iKM’s discrete diagnosis and histology fields, which are populated during the routine course of care (International Classification of Diseases codes were not used).  If no initial diagnosis date was documented, the first recorded diagnosis date in iKM was used. |
| Date of advanced/metastatic melanoma, advanced/metastatic NSCLC, or advanced/metastatic RCC diagnosis | iKM structured data | Eligibility criteria, Baseline characteristic | Prior medical history | Date of first recorded diagnosis of metastatic melanoma, metastatic NSCLC, or metastatic RCC within the iKM database. Specifically, the earliest associated date of any of these criteria:   1. Stage IV disease 2. TNM with M value of 1 3. Record of location of metastatic disease 4. Current or prior disease status containing reference to metastatic disease 5. A numbered LOT 6. A LOT with a reference to “metastatic”   To identify advanced status, the earliest associated date for Stage IIIB or Stage IIIC disease was used:   - Advanced melanoma = Stage III - Advanced NSCLC = Stage IIIB and Stage IIIC - Advanced/metastatic RCC = Stage IV |
| Time since initial diagnosis of melanoma, NSCLC, or RCC | iKM structured data | Baseline characteristic | Prior medical history | Duration of time, in weeks, between the date of melanoma, non small cell lung cancer, or RCC initial diagnosis and presentation of metastatic disease was calculated for each patient and summarized as mean (± SD) and median (range) of values. The number of patients with available data was reported.  Date of melanoma, NSCLC, or RCC cancer diagnosis and presentation of advanced or metastatic disease were determined as defined:   - Advanced melanoma = Stage III - Metastatic melanoma = Stage IV - Advanced NSCLC = Stage IIIB and Stage IIIC - Metastatic NSCLC = Stage IV - Advanced/metastatic RCC = Stage IV |
| Time since advanced/metastatic melanoma, advanced/metastatic NSCLC, or advanced/metastatic RCC diagnosis | iKM structured data | Baseline characteristic | Prior medical history | Duration of time, in weeks, between the index date and presentation of advanced/metastatic melanoma, advanced/metastatic NSCLC, or advanced/metastatic RCC was calculated for each patient and summarized as mean (± SD) and median (range) of values. The number of patients with available data is reported.  Presentation of advanced or metastatic disease was determined as defined:   - Advanced melanoma = Stage III - Metastatic melanoma = Stage IV - Advanced NSCLC = Stage IIIB and Stage IIIC - Metastatic NSCLC = Stage IV - Advanced/metastatic RCC = Stage IV |
| Follow-up Time | iKM structured data (derived) | Baseline characteristic | Follow-up | Duration (in months) between the index event and last visit date or death. |
| Distant metastatic site(s) | iKM structured data | Baseline characteristic | Baseline | Baseline metastatic location(s) were identified and categorized as: Bone; Brain; Liver; Lung; Lymph nodes; Other; Not documented (can indicate that metastases were not documented in the chart, not necessarily that patients did not have metastases.) |
| Count of metastatic site(s) | iKM structured data | Baseline characteristic | Baseline | Total count of metastatic site(s) at index: 1; 2; 3; 4+; No documentation (can indicate that metastases were not documented in the chart, and not necessarily that patients did not have metastases.)  Lymph node metastases were qualified as regional or distant. The “N” in TNM refers to regional node only. Metastasis to a non-regional i.e. distant node outside the draining basin of the appendage/ organ would be considered metastatic and listed with the “M”. E.g., T4 melanoma removed from the left arm with a positron emission tomography scan that showed mediastinal adenopathy, and a negative left axillary dissection is T4N0M1. |
| Stage at initial melanoma, NSCLC, or RCC diagnosis | iKM structured data | Baseline characteristic | Prior medical history | Categorized as: Stage 0; Stage IA; Stage IB; Stage IIA; Stage IIB; Stage IIIA; Stage IIIB; Stage IV; Not documented |
| TNM staging | iKM structured data | Baseline characteristic | Prior medical history | Patients’ stage was characterized according to TNM criteria recorded in the iKM EHR.  T-value at initial diagnosis: T0; T1; T1B; T2; T2A; T2B; T3; T4; TX – Tumor cannot be assessed  N-value at initial diagnosis: N0; N1; N2; NX – Node cannot be assessed  M-value at initial diagnosis: M0; M1 |
| Tumor grade | iKM structured data | Baseline characteristic | Prior medical history | Patients’ tumor grade was characterized as recorded in the iKM EHR. |
| Eastern Cooperative Oncology Group (ECOG) performance status | iKM structured data | Baseline characteristic | Baseline | ECOG performance status score is a rating of a patient’s disease status, daily living activities and quality of life, with low scores indicating greater functioning than high scores: 0; 1; 2; ≥3; Not documented  Karnofsky performance status is a similar measure and was converted to ECOG using the methodology outlined below.   \| **Karnofsky Performance Status** \| **ECOG Performance Status** \| **ECOG Performance Status Description** \| \| --- \| --- \| --- \| \| **100** \| 0 \| Fully active \| \| **80, 90** \| 1 \| Restricted in physically strenuous activity \| \| **60, 70** \| 2 \| Ambulatory and capable of self-care but unable to work \| \| **40, 50** \| 3 \| Capable only of limited self-care \| \| **10, 20, 30** \| 4 \| Completely disabled \| \| **0** \| 5 \| Dead \| \|  \|  \|  \| |
| Comorbidities | iKM structured data | Baseline characteristic | Baseline + Prior Medical History | Comorbidities documented within 6 months of the index date. These were captured as types and frequency. |
| Charlson Comorbidity Index (CCI) | iKM structured data (derived) | Baseline characteristic | Baseline | Categorized as: 0; 1; 2; 3+ |
| Hierarchical Condition Category (HCC) | iKM structured data (derived) | Baseline characteristic | Baseline | HCC risk adjustment score was captured if available in the problem list of the EHR and was queried from the structured data. |
| Disease histology (NSCLC) | iKM structured data | Baseline characteristic | Baseline | Categorized as: Adenocarcinoma; Adenosquamous carcinoma; Squamous cell carcinoma; Large cell carcinoma; Non-squamous cell carcinoma; Other; Not documented |
| PD-1 testing | iKM structured data | Baseline characteristic | Prior medical history | PD-1 testing status: Positive ≥ 1%; Negative < 1%; Pending/QNS; Documented unknown; No information |
| PD-L1 testing | iKM structured data | Baseline characteristic | Prior medical history | PD-L1 testing status: Positive ≥ 1%; Negative < 1%; Documented unknown; No information; Pending/quality or quantity not sufficient (QNS).  Note: Not routine for melanoma and RCC |
| *BRAF* mutation status | iKM structured data | Baseline characteristic | Prior medical history | BRAF status: Positive; Negative; Pending/QNS; Documented unknown; Not documented |
| *KRAS* mutation status | iKM structured data | Baseline characteristic | Prior medical history | KRAS status: Positive; Negative; Pending/QNS; Documented unknown; Not documented |
| *MET* mutation status | iKM structured data | Baseline characteristic | Prior medical history | MET status: Positive; Negative; Pending/QNS; Documented unknown; Not documented |
| Epidermal growth factor receptor (EGFR) mutation status | iKM structured data | Baseline characteristic | Prior medical history | EGFR status: Positive; Negative; Pending/QNS; Documented unknown; Not documented |
| ALK mutation status | iKM structured data | Baseline characteristic | Prior medical history | ALK status: Positive; Negative; Pending/QNS; Documented unknown; Not documented |
| ROS1 mutation status | iKM structured data | Baseline characteristic | Prior medical history | ROS1 status: Positive; Negative; Pending/QNS; Documented unknown; Not documented |
| Lactate dehydrogenase (LDH) | Structured | Baseline characteristic | Baseline (Lab) | Summarized as a continuous measure (i.e., with mean, standard deviation, median, Q1, Q3 and range)  When multiple values were available, the most recent entry was used. |
| Albumin | Structured | Baseline characteristic | Baseline (Lab) | Summarized as a continuous measure (i.e., with mean, standard deviation, median, Q1, Q3 and range)  When multiple values were available, the most recent entry was used. |
| C-reactive protein | Structured | Baseline characteristic | Baseline (Lab) | Summarized as a continuous measure (i.e., with mean, standard deviation, median, Q1, Q3 and range)  When multiple values were available, the most recent entry was used. |
| Calcium | Structured | Baseline characteristic | Baseline (Lab) | Summarized as a continuous measure (i.e., with mean, standard deviation, median, Q1, Q3 and range)  When multiple values were available, the most recent entry was used. |
| Complete blood count | Structured | Baseline characteristic | Baseline (Lab) | Laboratory results are presented as the mean (SD) and median (range), Q1 and Q3 and categorized as: Low; Normal; Elevated; Unknown  Normal lab values were categorized as:  White blood cell count (x 10^9^/L) = 3.4 - 9.6 x 10^9^/L  Neutrophils (x 10^9^/L) = 1.56 - 6.45 x 10^9^/L  Lymphocytes (x 10^9^/L) = 0.95 - 3.07 x 10^9^/L  Monocytes (x 10^9/L) = 0.26 - 0.81 x 10^9^/L  Eosinophil count (x 10^9^/L) = 0.03 - 0.48 x 10^9^/L  Basophils (x 10^9^/L) = 0.01-0.08 x 10^9^/L  Red blood cell count (x 10^12^/L)  Normal male range = 4.35 - 5.65 x 10^12^/L  Normal female range = 3.92 - 5.13 x 10^12^/L  Hemoglobin (g/dL)  Normal male range = 13.2 – 16.6 g/dL  Normal female range = 11.6 - 15.0 g/dL  Hematocrit (%)  Normal male range = 38.3% - 48.6%  Normal female range = 35.5% - 44.9%  Platelet count (x 10^9^/L)  Normal male range = 135 - 317 x 10^9^/L  Normal female range = 157 - 371 x 10^9^/L  **Individual labs are also reported for each.**  Reference ranges above are as reported by the Mayo Clinic <https://www.mayocliniclabs.com/test-catalog/index.html> |
| Anemia | iKM structured data (derived) | Baseline characteristic | Baseline (Lab) | Both hemoglobin and hematocrit were used to determine anemia status: defined as when both hemoglobin and hematocrit are below their normal levels (yes/no)  Normal lab values categorized as:  Hemoglobin (g/dL)  Normal male range = 13.2 – 16.6 g/dL  Normal female range = 11.6 - 15.0 g/dL  Hematocrit (%)  Normal male range = 38.3% - 48.6%  Normal female range = 35.5% - 44.9%  Reference ranges above are as reported by the Mayo Clinic  <https://www.mayoclinic.org/diseases-conditions/iron-deficiency-anemia/diagnosis-treatment/drc-20355040> |
| Absolute monocyte count (AMC; continuous) | iKM structured data (derived) | Baseline characteristic | Baseline (Lab) | AMC can be derived by multiplying total number of white blood cells in the body against percentage of white blood cells that are monocytes. For example, if the white blood cell count is 8000, and 5% of those white blood cells are monocytes, then the AMC would be 400 (8000 x 0.05).  Summarized as a continuous measure (i.e., with mean, standard deviation, median, and range)  When multiple values were available, the most recent entry was used. |
| Absolute monocyte count (AMC; categorical) | iKM structured data (derived) | Baseline characteristic | Baseline (Lab) | Categories of AMC collaboratively determined by Ontada and Partner Therapeutics, Inc. research teams were created based on the distribution of the continuous variable for absolute monocyte count (continuous) observed. |
| Absolute lymphocyte count (ALC; continuous) | iKM structured data (derived) | Baseline characteristic | Baseline (Lab) | ALC can be calculated by multiplying the total number of white blood cells against the percentage of white blood cells that are lymphocytes. If the white blood cell count is 8000, and 20% of those white blood cells are lymphocytes, that means the ALC would be 1600 (8000 x 0.2). |
| Absolute lymphocyte count (ALC; categorical) | iKM structured data (derived) | Baseline characteristic | Baseline (Lab) | Categories of ALC collaboratively determined by Ontada and Partner Therapeutics, Inc. research teams were created based on the distribution of the continuous variable for ALC (continuous) observed. |
| Monocyte to lymphocyte ratio (MLR; continuous) | iKM structured data (derived) | Baseline characteristic | Baseline (Lab) | MLR is defined as the AMC divided by the ALC |
| Monocyte to lymphocyte ratio (MLR; categorical) | iKM structured data (derived) | Baseline characteristic | Baseline (Lab) | Categories of MLR collaboratively determined by Ontada and Partner Therapeutics, Inc. research teams were created based on the distribution of the continuous variable for MLR (continuous) observed. |
| Absolute neutrophil count (ANC; continuous) | iKM structured data (derived) | Baseline characteristic | Baseline (Lab) | ANC can be calculated by multiplying the total number of WBCs by the percentage of neutrophils and dividing by 100. |
| Absolute neutrophil count (ANC; categorical) | iKM structured data (derived) | Baseline characteristic | Baseline (Lab) | Categories of ANC collaboratively determined by Ontada and Partner Therapeutics, Inc. research teams were created based on the distribution of the continuous variable for ANC (continuous) observed. |
| Neutrophil to lymphocyte ratio (NLR; continuous) | iKM structured data (derived) | Baseline characteristic | Baseline (Lab) | Defined as the ANC divided by the ALC |
| Neutrophil to lymphocyte ratio (NLR; categorical) | iKM structured data (derived) | Baseline characteristic | Baseline (Lab) | Categories of NLR collaboratively determined by Ontada and Partner Therapeutics, Inc. research teams were created based on the distribution of the continuous variable for NLR (continuous) observed. |
| International Metastatic RCC Database Consortium (IMDC) Risk Score for RCC | iKM structured data (derived) | Baseline characteristic | Baseline + Prior Medical History | Based on patient’s status of the following measures:   1. Performance status (Karnofsky score) < 80% (yes/no) 2. Time from diagnosis to systemic treatment < 12 months (yes/no) 3. Hemoglobin less than the lower limit of normal (yes/no) 4. Neutrophil > upper limit of normal (yes/no) 5. Calcium > upper limit of normal (yes/no) 6. Platelets > upper limit of normal (yes/no)   A Yes scores 1 point. The total IMDC score is based on the sum of scores of the 6 measures.  There were two options of reporting and the team chose between them when data were available, depending on the number of missing data:  1) The scores were calculated for patients who have all 6 measures available only  2) Due to incomplete documentation (not all 6 measures available) in the EHR, another option was to categorize patients into the following:  Confirmed-poor Risk: Patients who had three or more risk factors documented in the EHR.  Likely-Intermediate Risk: Patients who had one or two risk factors documented in the EHR (note, it is possible that some of these patients were poor risk, but the additional risk factors were not documented)  Favorable-Risk: Patients who had zero risk factors documented in the EHR  Unknown/Other Risk: Patients who had none of the risk factors documented in the EHR but who could not be assumed to be favorable because of insufficient documentation.  <https://www.imdconline.com/> |
| **Treatment characteristics** | | | | |
| Index treatment regimen | iKM structured data | Eligibility criteria, Treatment characteristics | Study observation period | Patients’ index treatment was categorized based on the cohort descriptions per advanced/metastatic tumor.  Melanoma   - Anti-PD-1 only (Nivolumab; Pembrolizumab - Anti-CTLA-4 only (Ipilimumab) - Anti-PD-1 + Anti-CTLA-4 (Nivolumab + Ipilimumab)   NSCLC   - Anti-PD-1 (Nivolumab; Pembrolizumab) - Anti-PD-L1 (Atezolizumab) - Anti-PD-1 + Anti-CTLA-4 (Nivolumab + Ipilimumab)   RCC   - Anti-PD-1 only (Nivolumab; Pembrolizumab) - Anti-PD-1 + Anti-CTLA-4 (Nivolumab + Ipilimumab) |
| Date of initiation of monotherapy or combination therapy of ipilimumab, pembrolizumab, nivolumab or atezolizumab during the patient identification period (i.e., index date) | iKM structured data | Eligibility criteria, Treatment characteristics | Study observation period | Date of initiation of monotherapy or combination therapy of ipilimumab, pembrolizumab, nivolumab or atezolizumab during the study identification period. If a regimen consisted of more than one drug with drugs given on different dates, the date of the first administration of any drug was used.  Patients who did not initiate a qualifying regimen during the patient identification period were excluded from the study. |
| Index treatment end date(s) | iKM structured data | Treatment characteristics | Study observation period | Date of final treatment for each drug or regimen. The patient’s treatment stop date may not have been documented if the patient died, was lost to follow-up or was still on therapy. The final treatment date, death date or end of study date was used, whichever was earliest. |
| Index line of therapy (LOT) | iKM structured data | Treatment characteristics | Study observation period | iKM includes a structured field to capture the LOT that is populated by providers when completing treatment orders. LOT indicators may have been incomplete or out-of-sequence for some patients. For these patients, programmatic logic was applied to categorize therapy sequences across LOTs based on start and stop-dates, as well as the predefined LOT indicator in iKM. LOT is categorized as: LOT1; LOT2; LOT3; LOT4; LOT5 |
| Index treatment by monocyte to lymphocyte ratio category (MLR; categorical) | iKM structured data (derived) | Treatment characteristics | Study observation period | A categorical variable containing both the patients’ index treatment and category of MLR was created.  Patients’ index treatment was categorized based on the cohort descriptions per Index treatment regimen (above) and by category of MLR.  Categories of MLR collaboratively determined by Ontada and Partner Therapeutics, Inc. research teams were created based on the distribution of the continuous variable for MLR (continuous) observed. |
| **Clinical outcomes** | | | | |
| Last US Oncology Network visit date | iKM structured data | Clinical outcomes | Study observation period | Each patient’s most recent visit date, prior to or on close of study observation period, in the structured data was recorded. A visit is defined as a physician encounter with the practice where either treatment is given or vital signs are recorded. For patients with a death date, this last visit date had to occur prior to the death date.  This is not reported separately; it was used in calculating the patient’s available follow-up time as a descriptive measure.  Available follow-up time = Integer (latest of last visit date or death date – index date +1) |
| Death date | iKM structured data + Limited Access Death Maste File (LADMF) | Clinical outcomes | Study observation period | Date of death was captured from the LADMF (https://dmf.ntis.gov) as well as iKM. When dates conflicted between the two sources, the LADMF date was prioritized. When severe data discordance was observed (i.e., death was reported to occur prior to the index date), then the iKM death date was used or if no additional death information was available, the discordant date was ignored. |
| Follow-up duration | iKM structured data + LADMF | Clinical outcomes | Study observation period | Duration of follow-up time (in months) from the index date. End of follow-up was defined as date of death, or among patients who did not die, last visit date with vital signs or end of the study period, whichever occurred first.  Data were summarized as a continuous measure (i.e., with mean, standard deviation, median and range). |
| Vital status | iKM structured data + LADMF | Clinical outcomes | Study observation period | Patients without a date of death in either the LADMF or iKM were assumed to be alive at the end of the study. Those with a date of death in either LADMF or iKM were flagged as deceased. |
| Overall survival (OS) | iKM structured data + LADMF | Clinical outcomes | Study observation period | OS was defined as the interval (in weeks) between the index treatment and the date of death (any cause) as documented in the LADMF and the iKM EHR database. Patients who did not die within the study observation period were censored on the study end date or the last visit date available in the dataset, whichever occurred first. OS was analyzed using the Kaplan-Meier method with 95% Cis. |
| Time to treatment discontinuation (TTD) | iKM structured data + LADMF | Clinical outcomes | Study observation period | TTD was defined as the interval (in weeks) between the initiation of the index treatment and discontinuation for any cause. Patients who did not discontinue treatment during the study observation period were censored on the study end date or the last visit date available in the dataset, whichever occurred first. TTD was analyzed using the Kaplan-Meier method with 95% CIs. |
| Time to next treatment (TTNT) | iKM structured data + LADMF | Clinical outcomes | Study observation period | TTNT was measured from initiation of the index treatment to the start date of the next treatment or date of death due to any cause. Patients who do not receive a subsequent treatment and were alive at the end of the study observation period were censored on the study end date or the last visit date available in the dataset, whichever occurred first. TTNT was estimated in weeks using the Kaplan-Meier method with 95% CIs. |

**Statistical Analysis**

Impacts of baseline biomarkers on treatment outcomes were estimated by hazard ratios (HR) with 95% CI. Time-to-event outcomes were assessed by Kaplan-Meier methods with 95% CI. Log-rank tests of survival curves informed whether time-to-event variables were different for each cancer type by subgroups and levels of AMC or MLR. Distributions of baseline characteristics across subgroups were compared by chi-square test or Fisher’s exact test for categorical variables and either t-test or Kruskal-Wallis test for continuous variables.

Multivariable Cox regression models were adjusted for confounders and important indicators at baseline by cancer type. Multivariable Cox proportional hazard regression models were used to assess associations among selected variables and OS, TTD, and TTNT for each cancer type. Patients with missing values for confounders and important indicators were modelled as a separate category and included in the final multivariable model. Missing values for a particular variable beyond 50% in univariate analyses were excluded from multivariable Cox regression models.

**Supplemental Results**

**Supplement Table S2.** Biomarkers

| **Biomarkers** | **Melanoma**  n=3314 | **NSCLC**  n=12416 | **RCC**  n=2456 |
| --- | --- | --- | --- |
| **PD-L1 – n (%)** |  |  |  |
| Positive (≥ 1 expression) | 0 | 3682 (29.65) | 2 (0.08) |
| Negative | 0 | 1572 (12.66) | 0 |
| Not documented | 3314 (100.00) | 7154 (57.62) | 2454 (99.91) |
| **EGFR – n (%)** |  |  |  |
| Positive | 1 (0.00) | 404 (3.25) | 0 |
| Negative | 1 (0.00) | 5221 (42.10) | 1 (0.00) |
| Equivocal | 0 | 19 (0.15) | 0 |
| Not documented | 3312 (99.99) | 6765 (54.49) | 2455 (99.95) |
| **ALK – n (%)** |  |  |  |
| Positive | 0 | 54 (0.43) | 0 |
| Negative | 1 (0.00) | 5483 (44.16) | 1 (0.00) |
| Equivocal | 0 | 60 (0.48) | 0 |
| Not documented | 3313 (100.00) | 6806 (54.82) | 2455 (99.95) |
| **ROS1 – n (%)** |  |  |  |
| Positive | 0 | 33 (0.27) | 0 |
| Negative | 1 (0.00) | 3982 (32.07) | 0 |
| Not documented | 3313 (100.00) | 8396 (67.62) | 2456 (100.00) |
| **BRAF – n (%)** |  |  |  |
| Positive | 490 (14.78) | 100 (0.81) | 0 |
| Wild-type/Negative | 777 (23.44) | 2475 (19.93) | 0 |
| Not documented | 2043 (61.64) | 9839 (79.24) | 2456 (100.00) |
| **MET – n (%)** |  |  |  |
| Positive | 0 | 1 (0.00) | 0 |
| Negative | 0 | 269 (2.17) | 0 |
| Not documented | 3314 (100.00) | 12141 (97.79) | 2456 (100.00) |
| **KRAS – n (%)** |  |  |  |
| Positive | 0 | 70 (0.56) | 1 (0.0) |
| Wild-type | 23 (0.69) | 34 (0.27) | 0 |
| Not documented | 3291 (99.30) | 12312 (99.16) | 2455 (99.95) |

ALK: anaplastic lymphoma kinase; BRAF: B-Raf proto-oncogene; EGFR: epidermal growth factor receptor; KRAS: KRAS proto-oncogene; MET: mesenchymal epithelial transition; NSCLC: non-small cell lung cancer; PD-L1: programmed death ligand-1; RCC: renal cell carcinoma; ROS1: c-ros oncogene 1

**Supplement Table S3.** Univariate Cox model for NLR on OS

|  | **Melanoma** | | **NSCLC** | | **RCC** | |
| --- | --- | --- | --- | --- | --- | --- |
|  | **p-value** | **HR (95% CI)** | **p-value** | **HR (95% CI)** | **p-value** | **HR (95% CI)** |
| **Elevated^*^**  **NLR** | p<0.0001 | 2.81  (2.47, 3.20) | p<0.0001 | 1.68  (1.58, 1.77) | p<0.0001 | 1.96  (1.72, 2.23) |
| **Low** | p=0.353 | 1.77  (1.04, 3.02) | p=0.8511 | 1.03  (0.73, 1.46) | p=0.8303 | 0.926  (0.46, 1.87) |
| **Not documented** | p=0.0876 | 1.32  (0.96, 1.80) | p<0.0001 | 1.28  (1.15, 1.42) | p=0.6704 | 0.93  (0.65, 1.32) |

HR: hazard ratio; NLR: neutrophil-to-lymphocyte ratio; NSCLC: non-small cell lung cancer; RCC: renal cell carcinoma

^*^Normal NLR: 1-2 (Zahorec R. Neutrophil-to-Lymphocyte Ratio, Past, Present and Future Perspectives. *Bratislava Medical Journal* (2021) 122(07):474-88. doi: 10.4149/bll_2021_078.)

Summarized data from **Figure 4. Forest plots of adjusted hazard ratios on overall survival by tumor type**:

*Covariate data that correlated with longer or decreased OS can be found in the supplement.*  albumin concentration per unit increase (melanoma HR 0.43; NSCLC HR 0.61; RCC HR 0.57, all p<0.0001); eosinophil count (melanoma Q2–5, HR 0.53, 0.54, 0.53, 0.45, p<0.0001; NSCLC Q3–5,HR 0.90, p=0.022; HR 0.76, p<0.0001; HR 0.73, p< 0.0001; and RCC Q3–Q5, HR 0.58, p<0.0001; HR 0.61, p< 0.0001; HR 0.68, p=0.002).

*Covariates of interest correlated with decreased OS include*: Northeast and undocumented regions (melanoma HR 1.61, p=0.004; HR 17.11, p< 0.0001); Midwest and undocumented regions (NSCLC HR 1.22, p< 0.0001; HR 2.47, p=0.0105); Midwest, Northeast, and undocumented regions (RCC HR 1.25, p=0.0215, HR 1.47, p=0.0326, and HR 0.000, p< 0.0001).MLR (melanoma: Q4-Q5, HR 1.46, p=0.0035; HR 1.38, p=0.0225; NSCLC: Q2-Q5, HR 1.16, p=0.0023; HR 1.20, p=0.0001; HR 1.28, p< 0.0001; HR 1.45, p< 0.0001; RCC: Q3-Q5, HR 1.30, p=0.0278; HR 1.36, p=0.011; HR 1.60, p=0.0003).

**Supplement Figure S1.** OS, TTD, and TTNT survival per treatment class


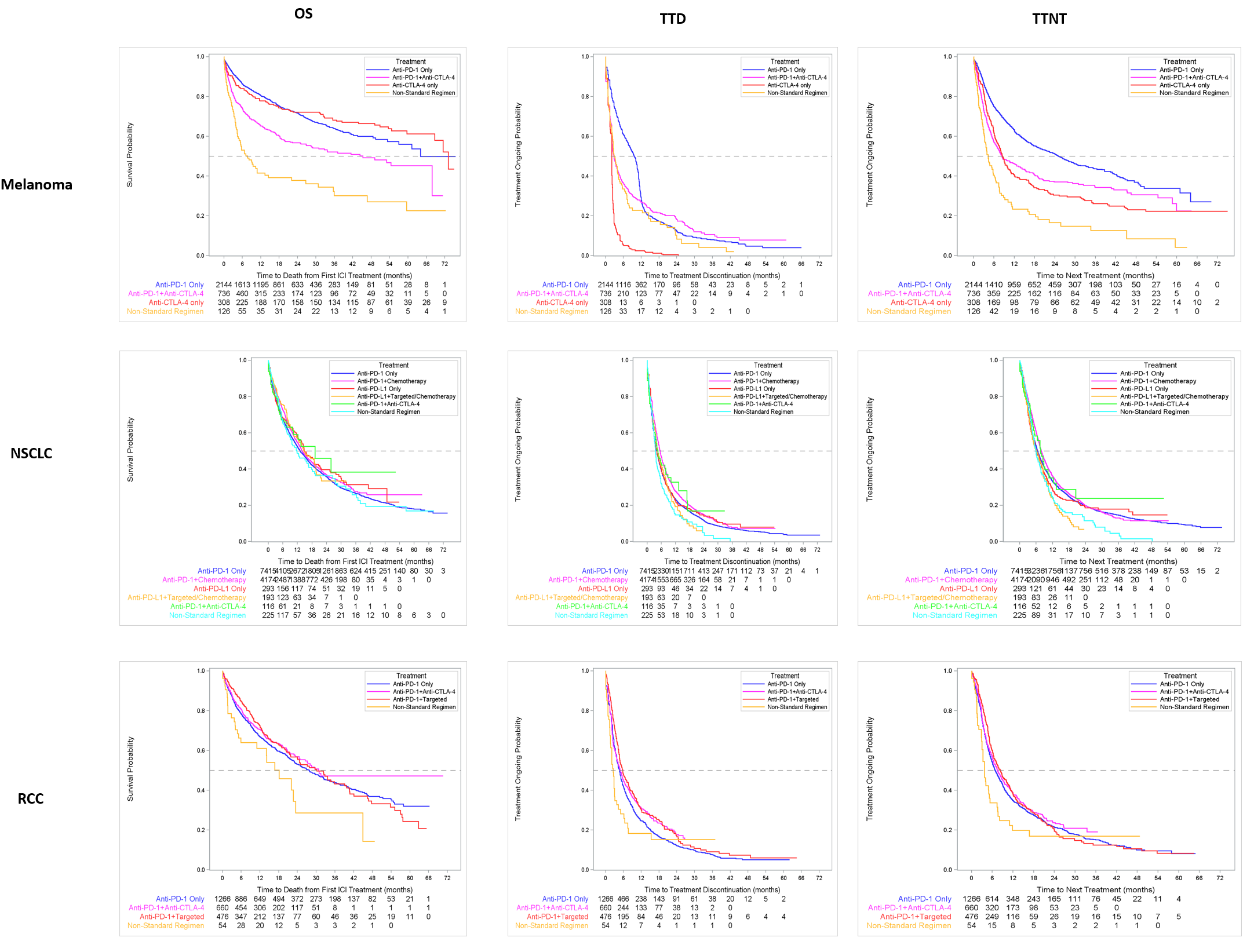


NSCLC: non-small cell lung cancer; OS: overall survival; RCC: renal cell carcinoma; TTD: time to discontinuation; TTNT: time to next treatment

**Time to Treatment Discontinuation**

Longest median (95% CI) time to treatment discontinuation (TTD) for melanoma, NSCLC, and RCC was observed with anti-PD-1 monotherapy (9.70 [9.00 - 10.20] months), anti-PD-1 + chemotherapy (5.60 [5.20 -5.70] months), and anti-PD-1 + targeted therapy (5.70 [4.90 -6.80] months), respectively. Longest median (95% CI) TTD was observed for lower AMC quintiles(Q) (melanoma AMC Q2, 8.10 [6.80 – 9.50] months; NSCLC AMC Q1 and AMC Q2, 5.10 [4.70 – 5.40] months and 5.10 [4.80 - 5.60] months, respectively; RCC AMC Q2, 5.30 [4.60 - 6.40] months). Similarly, TTD trends for ANC were associated with longer median TTD (95% CI) in lower ANC quintiles (melanoma ANC Q1, 8.50 [6.90 – 10.20] months; NSCLC Q1, 5.40 [5.00 – 5.80] months; RCC Q2, 5.70 [5.00-6.80] months). Lower MLR quintiles were associated with longest median (95% CI) TTD (melanoma MLR Q2, 8.40 [6.90 - 10.20] months; NSCLC MLR Q1, 6.20 [5.60 - 6.50]; RCC MLR Q2, 5.80 [4.70 - 6.50] months).

**Supplement Figure S2.** TTD survival per AMC, ANC, MLR


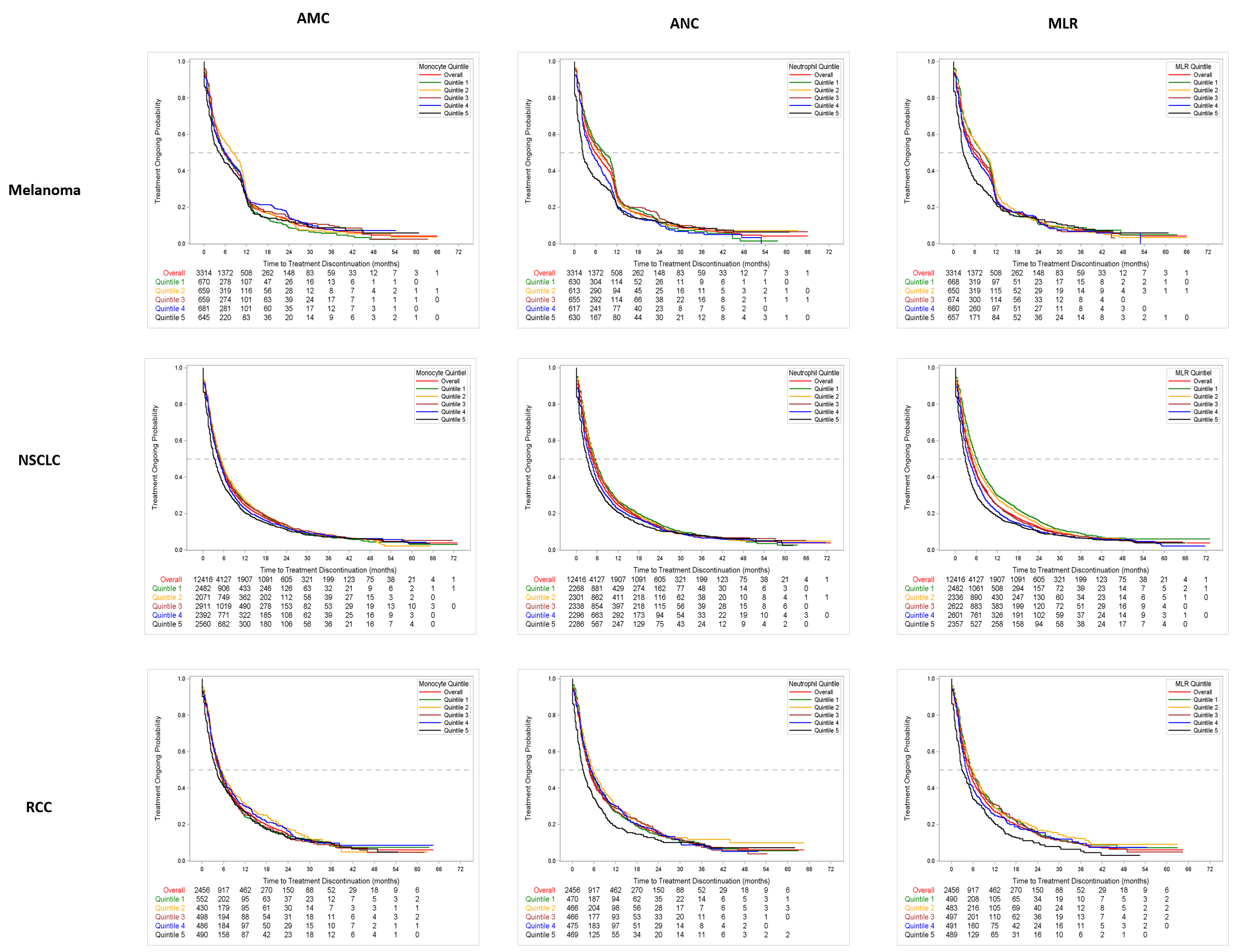


AMC: absolute monocyte count; ANC: absolute neutrophil count; MLR: monocyte-to-lymphocyte ratio; NSCLC: non-small cell lung cancer; RCC: renal cell carcinoma

**Univariate Analyses**

Longest median OS for the melanoma cohort was with AMC Q1 (median [95% CI] 71.40 [59.50 – not reached]). Longest median OS for the NSCLC cohort was with AMC Q2 and Q3 (15.90 [14.40 – 18.00] months and 15.90 [14.80 – 17.10] months, respectively). Lastly for the RCC cohort, longest median OS was with AMC Q2 (37.50 [30.90 – 44.70] months). Differences among AMC quintile median OS durations compared to Q1 include melanoma AMC Q4-5 (HR 1.25, p=0.028; HR 1.90, p<0.0001), NSCLC AMC Q4-5 (HR 1.18, p<0.0001; HR 1.48, p<0.0001), and RCC AMC Q5 (HR 1.46, p<0.0001).

In the melanoma cohort, longest median OS was with ANC Q3 (73.10 [53.30 – not reached] months), whereas for NSCLC and RCC cohorts median OS was longest with ANC Q1 (NSCLC, 19.30 [17.10 – 21.70] months; RCC, 34.70 [28.60 – 43.40] months). Differences among ANC quintile median OS durations compared to Q1 include melanoma ANC Q3-5 (HR 1.33, p=0.013; HR 1.90, p<0.0001; HR 3.31, p<0.0001), NSCLC ANC Q2-5 (HR 1.13, p<0.0029; HR 1.23, p<0.0001; HR 1.51, p<0.0001; HR 1.91, p<0.0001), and RCC ANC Q5 (HR 1.94, p<0.0001).

In the melanoma cohort, longest median OS was with MLR Q2 (73.10 [61.20 – not reached] months), whereas in NSCLC and RCC cohorts median OS was longest for MLR Q1 (NSCLC, 22.30 [20.20 – 24.70] months; RCC, 44.90 [37.20 – 55.10] months). Differences among MLR quintile median OS durations compared to Q1 include melanoma MLR Q3-5 (HR 1.40, p=0.0031; HR 2.20, p<0.0001; HR 3.26, p<0.0001), NSCLC MLR Q2-5 (HR 1.16, p=0.0004; HR 1.41, p<0.0001; HR 1.68, p<0.0001; HR 2.21, p<0.0001), and RCC MLR Q3-5 (HR 1.36, p=0.0038; HR 1.79, p<0.0001; HR 2.67, p<0.0001).

Unlike AMC and MLR, for all three cohorts, longest OS was observed in the higher eosinophil count quintiles as compared to Q1. Longest OS in the melanoma cohort was observed in eosinophil count Q3 (73.10 (NR – NR) months) and in eosinophil count Q4 in the NSCLC and RCC cohorts (NSCLC, 16.60 [15.00 - 18.10] months; RCC, 40.70 [29.60 – 57.70] months). Eosinophil quintile associations found when compared to Q1 include melanoma Q2-5 (HR 0.423, p<0.0001; HR 0.39, p<0.0001; HR 0.41, p<0.00001; HR 0.34, p<0.0001), NSCLC Q2-5 (HR 0.80, p<0.0001; HR 0.76, p<0.0001; HR 0.65, p<0.0001; HR 0.67, p<0.0001), and RCC Q2-5 (HR 0.73, p=0.0004; HR 0.50, p<0.0001; HR 0.50, p<0.0001; HR 0.51, p<0.0001).

**Supplement Figure S3.** OS, TTD, and TTNT survival for Eosinophil Count


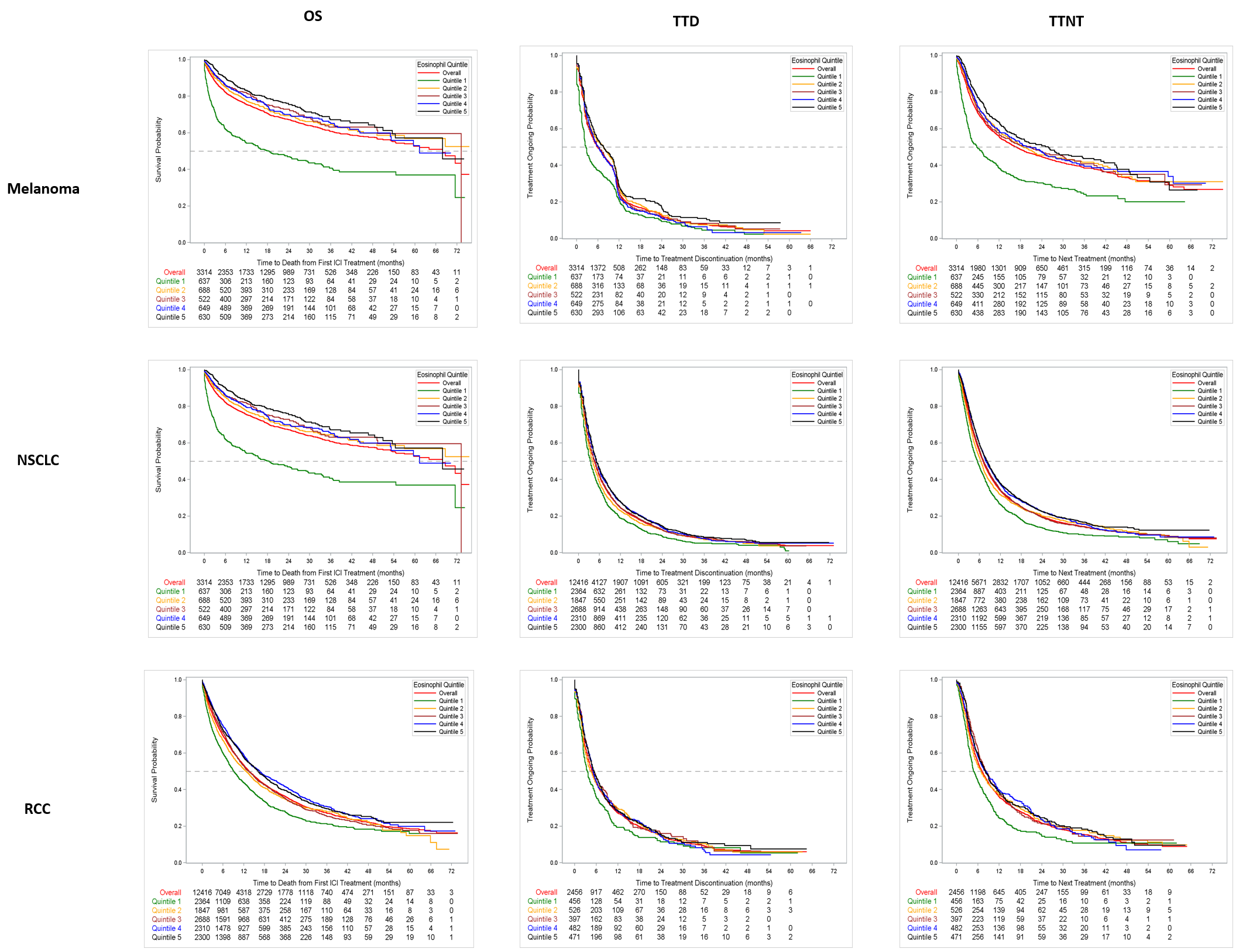


NSCLC: non-small cell lung cancer; OS: overall survival; RCC: renal cell carcinoma; TTD: time to discontinuation; TTNT: time to next treatment

**Time to Next Treatment**

Shortest median (95% CI) time to next treatment (TTNT) was observed among the non-standard regimen group in all three tumor types (melanoma, 4.20 [3.30 - 5.80]; NSCLC, 5.90 [5.10 - 7.20], RCC, 3.70 [2.90 - 5.10] months). Shortest median (95% CI) TTNT was observed in Q5 AMC in all three tumor types (melanoma AMC Q5, 10.20 [8.00 – 12.70] months; NSCLC AMC Q5, 5.30 [4.90 - 5.60] months; RCC AMC Q5 6.00 [5.10 – 7.40] months). Similarly, TTNT trends for ANC Q5 was associated with shortest median (95% CI) TTNT (melanoma ANC Q5, 5.70 [4.60 - 7.40] months; NSCLC ANC Q5, 4.70 [4.40 – 5.20] months; RCC ANC Q5, 4.70 [4.20 – 5.60] months). MLR Q5 was associated with shortest median (95% CI) TTNT in all three tumor types (melanoma MLR Q5, 5.60 [4.70 - 6.50] months; NSCLC MLR Q5, 4.50 [4.20 – 4.80]; RCC MLR Q5, 4.80 [3.90 – 5.60] months). Conversely eosinophil Q1 was associated with shortest median (95% CI) TTNT in all three tumor types (melanoma eosinophil Q1, 5.70 [4.20 – 7.60] months; NSCLC eosinophil Q1, 5.40 [5.00 – 5.80] months; RCC eosinophil Q1, 4.80 [4.40 – 5.90] months).

**Supplement Figure S4.** TTNT survival per AMC, ANC, MLR


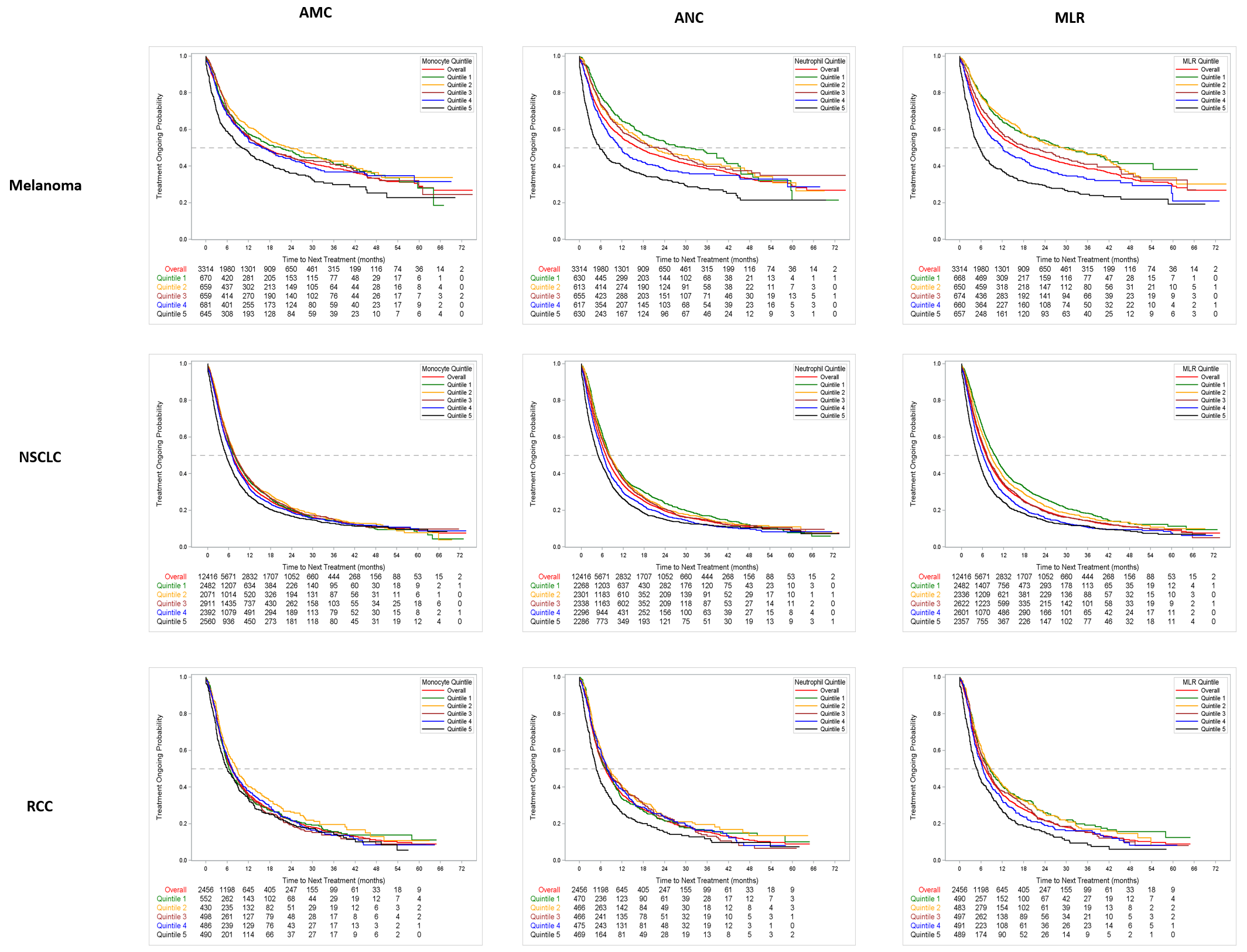


AMC: absolute monocyte count; ANC: absolute neutrophil count; MLR: monocyte-to-lymphocyte ratio; NSCLC: non-small cell lung cancer; RCC: renal cell carcinoma

**Supplement Figure S5.** Overall Survival per tumor type for AMC, ANC, ALC, Eosinophil Count


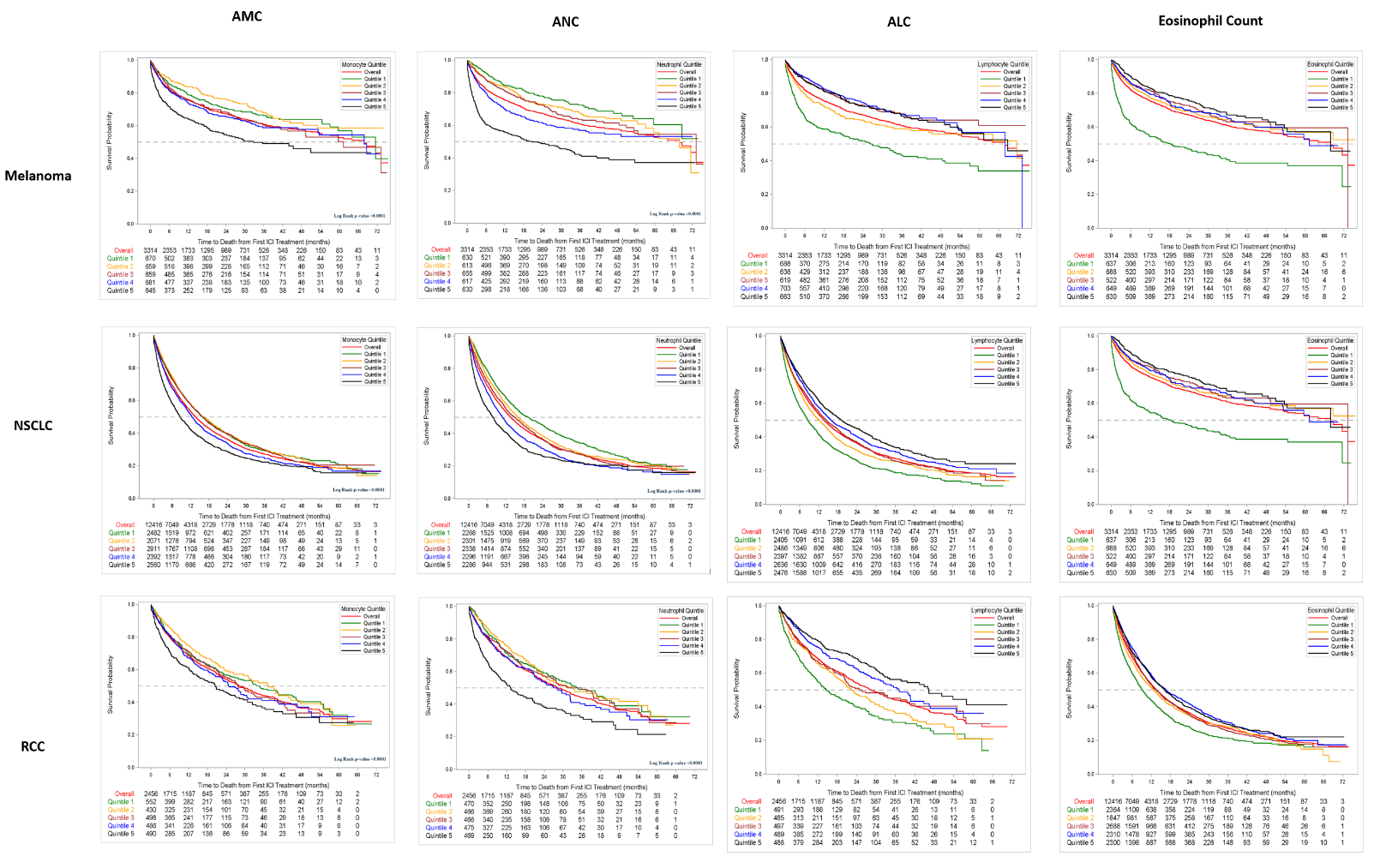


ALC: absolute lymphocyte count; AMC: absolute monocyte count; ANC: absolute neutrophil count; MLR: monocyte-to-lymphocyte ratio; NSCLC: non-small cell lung cancer; RCC: renal cell carcinoma
