# Supplementary material for: Real‐world outcomes of 18,186 metastatic solid tumor outpatients: Baseline blood cell counts correlate with survival after immune checkpoint inhibitor therapy
Source: Cancer Med. 2023 Nov 14;12(22):20783–97. doi: 10.1002/cam4.6645 (PMC10709745; doi:10.1002/cam4.6645)
Supplement: Supplementary file 3 — Media Information: Video 1. [file CAM4-12-20783-s002.docx]

| **Media Information** | |
| --- | --- |
| **Title** | Video 1 |
| **Caption** | Dr. Jerome Goldschmidt, Physician Investigator at The US Oncology Network, describes the baseline survival indicators found in the retrospective observational study of over 18,000 adults with advanced melanoma, non-small cell lung cancer, or renal cell carcinoma treated with immune checkpoint inhibitors. |
| **Placeholder image** | 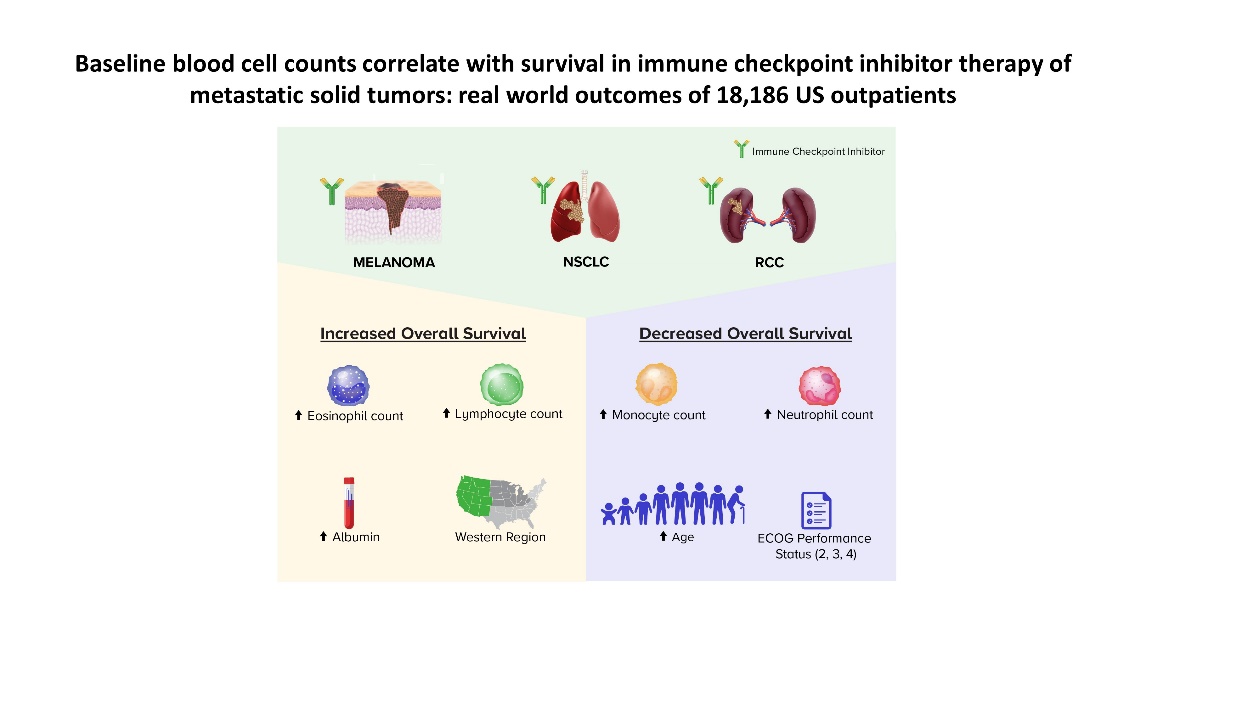 |
| **Transcript** | Submit as separate Word document |
| **Alt text** | Transcript below |

**Transcript**

Immune checkpoint inhibitor therapy has improved patient survival in multiple advanced or metastatic cancers, however there is still work to be done to establish biomarkers for prognosis and treatment selection.

In our retrospective observational study, we analyzed demographic, clinical, laboratory, and treatment data of over 18,000 adults treated at outpatient clinics within The US Oncology Network. Patients were included if they were diagnosed with advanced or metastatic melanoma, non-small cell lung cancer, or renal cell carcinoma between January 2015, and the end of November 2020 and were given immune checkpoint inhibitor monotherapy or combination therapy with ipilimumab, pembrolizumab, nivolumab, or atezolizumab.

Treatment outcomes including overall survival were followed until the end of May 2021, last patient record, or date of death.

Better overall survival correlated with increased baseline serum albumin, increased eosinophil and lymphocyte count, and Western United States physician practice location. Whereas, decreased overall survival correlated with increased baseline monocyte count, monocyte-to-lymphocyte ratio, neutrophil count, age, and worse ECOG Performance Status.

This study is the largest to date to associate easily obtainable baseline survival indicators and outcomes in patients with these tumors receiving immune checkpoint inhibitors. Results may inform prognostic models and equip providers with a practical way to implement precision medicine to identify patients most likely to benefit from immune checkpoint inhibitor therapy.
